# Mixed‐Precision for Linear Solvers in Global Geophysical Flows

**DOI:** 10.1029/2022MS003148

**Published:** 2022-09-10

**Authors:** Jan Ackmann, Peter D. Dueben, Tim Palmer, Piotr K. Smolarkiewicz

**Affiliations:** ^1^ University of Oxford Oxford UK; ^2^ European Centre for Medium Range Weather Forecasts Reading UK; ^3^ National Center for Atmospheric Research Boulder CO USA

**Keywords:** high performance computing, reduced precision, linear solver, semi‐implicit timestepping, uncertainty quantification

## Abstract

Semi‐implicit (SI) time‐stepping schemes for atmosphere and ocean models require elliptic solvers that work efficiently on modern supercomputers. This paper reports our study of the potential computational savings when using mixed precision arithmetic in the elliptic solvers. Precision levels as low as half (16 bits) are used and a detailed evaluation of the impact of reduced precision on the solver convergence and the solution quality is performed. This study is conducted in the context of a novel SI shallow‐water model on the sphere, purposely designed to mimic numerical intricacies of modern all‐scale weather and climate (W&C) models. The governing algorithm of the shallow‐water model is based on the non‐oscillatory MPDATA methods for geophysical flows, whereas the resulting elliptic problem employs a strongly preconditioned non‐symmetric Krylov‐subspace Generalized Conjugated‐Residual (GCR) solver, proven in advanced atmospheric applications. The classical longitude/latitude grid is deliberately chosen to retain the stiffness of global W&C models. The analysis of the precision reduction is done on a software level, using an emulator, whereas the performance is measured on actual reduced precision hardware. The reduced‐precision experiments are conducted for established dynamical‐core test‐cases, like the Rossby‐Haurwitz wavenumber 4 and a zonal orographic flow. The study shows that selected key components of the elliptic solver, most prominently the preconditioning and the application of the linear operator, can be performed at the level of half precision. For these components, the use of half precision is found to yield a speed‐up of a factor 4 compared to double precision for a wide range of problem sizes.

## Key Points

- A detailed study of mixed‐precision for linear solvers for weather and climate models is performed, including half precision with 16 bits
- Precision can be reduced in important parts of the solver but a naive approach to reduce precision everywhere does not work
- Compute time can be reduced by a factor four compared to double precision for parts that work in half precision

## 1 Introduction

In the simulation of complex multi‐scale flows arising in weather and climate (W&C), one of the biggest challenges is to satisfy strict service requirements in terms of time‐to‐solution and to satisfy budgetary constraints in terms of energy‐to‐solution, without compromising the accuracy and stability of the application (Müller et al., 2019). One way to tackle this challenge is to use reduced‐precision arithmetic in W&C models. The interest with reduced precision or the combination of different precision levels in ”mixed precision” approaches has already some history in scientific computation (Baboulin et al., 2009; Chen et al., 2012; Furuichi et al., 2011; Göddeke et al., 2007). However, it is relatively new in W&C modeling. This effort started with (Palmer, 2012) and (Düben et al., 2014) where reduced precision was motivated by the large degree of uncertainty that is present in W&C models due to their nonlinear dynamics and high level of complexity which makes it difficult to justify high numerical precision. The study of mixed precision in numerical modeling is timely, since the recent boom of machine learning methods is pushing developments of supercomputing hardware toward very efficient dense linear algebra performed at low precision. This hardware is specialized for the use in deep learning where half precision arithmetic—or even less—is often sufficient; see for example, the Tensor Processing Unit (TPU) by Google (Jouppi et al., 2017).

A large part of W&C models is used to represent subgrid scale processes—e.g., turbulence and cloud physics—that carry a large degree of inherent uncertainty (Saffin et al., 2020). In principle one can thus argue that representation of such processes is the prime candidate for a precision reduction. The other dominant part of W&C models is the dynamical core (a.k.a dycore) which provides discrete solutions to the Navier‐Stokes equations. Although there is little uncertainty about the equations per se, their numerical solution procedures introduce model error that add uncertainty to the dynamics. Early work on thorough analysis to identify the minimal level of numerical precision that can be used in different parts of dycores has focused on spectral model formulations (Chantry et al., 2019; Düben & Palmer, 2014). Similar work on grid‐point model formulations has only just begun with the use of single precision arithmetic (Maynard & Walters, 2019; Nakano et al., 2018; Tinto Prims et al., 2019) with 32 bits per variable.

In efficient W&C dycores the semi‐implicit (SI) timestepping is typically used for the atmospheric (Mengaldo et al., 2019) as well as the oceanic (Adcroft et al., 2004; Korn, 2017; Wang et al., 2014) component. Its key virtue is extended computational stability with respect to acoustic, buoyant and rotational modes of motion allowing for the use of relatively long time steps. On the other hand, the approach results in an intricate linear problem and complicated elliptic boundary value problem for pressure (viz. Schur complement). In particular, the SI approach is at the heart of the newly developed finite‐volume module (FVM) of the Integrated Forecasting System (IFS) at the European Centre for Medium‐Range Weather Forecasts (ECMWF) (Kühnlein et al., 2019; Smolarkiewicz et al., 2016, 2017, 2019). The FVM provides an alternative dynamical core to the spectral transform based IFS. The two essential ingredients of the FVM timestepping is the Multidimensional Positive Definite Advection Transport Algorithm (MPDATA) approach (Kühnlein & Smolarkiewicz, 2017; Smolarkiewicz & Szmelter, 2005; Szmelter & Smolarkiewicz, 2010) and the bespoke preconditioned nonsymmetric Krylov‐subspace elliptic solver (Kühnlein et al., 2019) built on the Generalized Conjugated‐Residual (GCR) approach of (Eisenstat et al., 1983). While MPDATA controls the explicit part of the timestepping, GCR inverts the Schur complement of the linear problem.

Undeniably, elliptic solvers are computationally demanding and substantial development has been invested into making them as efficient as possible for W&C models on modern supercomputers (Kühnlein et al., 2019; Mueller & Scheichl, 2014; Yang et al., 2016). To efficiently solve an elliptic problem posed in a thin spherical shell (such as the global atmosphere) ultimately requires matrix inversion—if not in the main solver than at least in its preconditioner. While there is the common opinion that high precision is required for this purpose, there are theoretical studies (Anzt et al., 2019; Baboulin et al., 2009; Carson & Higham, 2018; Göddeke et al., 2007; Haidar et al., 2018) suggesting that mixed‐precision approaches could be exploited, and there are already some applications of mixed‐precision elliptic solvers in CFD (Amritkar & Tafti, 2016; Furuichi et al., 2011; Idomura et al., 2018) and also in W&C (Maynard & Walters, 2019). Although these approaches may differ in choice of algorithms and applications, a common theme emerges that the preconditioning step may be a good choice for reducing precision, where some work goes as low as half precision arithmetic representing each real number with only 16 bits (as a floating point number) and decimal precision reduced to three digits.

The aim of this paper is to establish the limits of precision for the specific combination of characteristics that make SI W&C models unique, while its objective is to perform an in‐depth analysis of the behavior of mixed‐precision elliptic solvers in such an environment. The elliptic problems encountered in SI W&C models are non‐symmetric, and are typically solved using iterative solvers due to the enormous problem size. We solve a fluid dynamics problem on the sphere, which comes with grid irregularities, giving rise to a specific structure of the involved linear operator. In effect, a standard classification of the problem based solely on the operator condition number does not give full justice to the complexity of the problem; cf. (Kühnlein et al., 2019; Smolarkiewicz et al., 2019). Moreover, the elliptic problem is part of an entire SI timestepping scheme and as such is in a feedback loop with the non‐linear dynamics of the model. Consequently, we solve much more at each time step than a sole elliptic problem. Different solver solutions, even obtained to the same accuracy in terms of the residual errors, can lead to vastly different behavior and model error growth throughout a simulation. For these reasons, our mixed‐precision elliptic solver needs to be tailored to the specific application at hand and thoroughly tested in this specific W&C environment.

Herein, we study the impact of mixed‐precision in the elliptic solver for W&C models by investigating a representative SI shallow‐water model on the sphere and using the MPDATA approach. With these choices, we stay conceptionally close to the ideas employed in the IFS‐FVM. The latter is formulated in the latitude‐longitude (lat‐lon) coordinate framework, but circumvents polar grid singularity via a quasi‐uniform unstructured mesh discretization, whereby the essential problem stiffness comes from the thin vertical dimension. The shallow‐water model of this paper uses a regular lat‐lon grid subject to polar singularity (Prusa, 2018), but retains the aspect of the problem stiffness which is coming from the longitudinal dimension. Given the stiffness and grid singularity that are already prone to creating model errors in double precision (DP) arithmetic (64 bits per variable), we carefully study what happens to solutions in polar regions under reduced precision. We test our mixed‐precision solver on test‐cases from the well‐known test‐suite for shallow‐water dycores (Williamson et al., 1992). The first test‐case is the standard problem of the Rossby‐Haurwitz Wave with wave number 4. The second test‐case paraphrases the standard zonal flow over an isolated mountain by replacing the idealized smooth hill with the natural orography of the Earth.

In this paper, whenever we refer to mixed‐precision, we mean the use of different precision levels within the same simulation. This can either refer to the use of single and DP as available on modern computers, or the use of half, single and DP. While half precision is available on machine learning accelerators on modern supercomputing hardware, it is currently not yet available for use in most high‐level computer languages. An important milestone in this regard is that Fugaku, number one supercomputer on the TOP 500 list for two years (June 2020‐June 2022; https://www.top500.org/system/179807/), allows for the use of half precision arithmetic from Fortran. For this paper, half precision will be emulated in software as well as used on actual hardware, here on the Fujitsu A64FX processors (Odajima et al., 2020) designed for Fugaku which are also available on the supercomputer Isambard 2. The reduced‐precision emulator is used because it enables to easily explore different precision levels, while the ensuing performance measurements are performed on Isambard 2.

The paper is structured as follows. In Section 2, we describe the continuous as well as discretized model and introduce our elliptic solver and the preconditioner. Section 3 presents the test‐cases, the model setup and a description of the reduced‐precision experiment suite. In Section 4 we show and discuss reduced‐precision results. Section 5 concludes the paper.

## 2 Model Description

### 2.1 The Governing Equations

With our choice of model, we aim to stay conceptionally as close as possible to the approach used by other MPDATA based fluid models (Kurowski et al., 2016; Prusa et al., 2008; Szmelter & Smolarkiewicz, 2010) and consequently the newly developed IFS‐FVM atmospheric model (Kühnlein et al., 2019; Smolarkiewicz et al., 2016, 2019). Thus, we chose to use the shallow‐water equations (SWE) on the sphere written in the form of generalized transport equations (Smolarkiewicz & Margolin, 1998; Szmelter & Smolarkiewicz, 2010):

(1) $$\frac{\partial G\Phi }{\partial t}+\nabla \cdot \left(v\Phi \right)=0,$$

(2) $$\frac{\partial GQ_{x}}{\partial t}+\nabla \cdot \left(vQ_{x}\right)=G\;R_{x},$$

(3) $$\frac{\partial GQ_{y}}{\partial t}+\nabla \cdot \left(vQ_{y}\right)=G\;R_{y},$$

where Φ is the fluid thickness, ∇ = (*∂*/*∂x*, *∂*/*∂y*), $Q_{x}=\Phi \;\dot{x}h_{x}$ and $Q_{y}=\Phi \;\dot{y}h_{y}$ denote the momenta in *x* (longitudinal) and *y* (latitudinal) directions, while *G* = *h*_*x*_ *h*_*y*_ is the Jacobian of the geospherical framework with *h*_*x*_, *h*_*y*_ being the metric coefficients of the general orthogonal coordinates. Here, *h*_*x*_ = *a* cos(*ϕ*), *h*_*y*_ = *a* for a lat‐lon grid with Earth's radius *a*, so longitude *λ* = *x* and latitude *ϕ* = *y*. The advective velocity **v** relates to the momentum **Q** = (*Q*_*x*_, *Q*_*y*_) via

(4) $$v=\left(\dot{x},\;\dot{y}\right)\;G=\left(Q_{x}h_{y},\;Q_{y}h_{x}\right)/\Phi \;;$$

cf. (Smolarkiewicz & Margolin, 1998; Szmelter & Smolarkiewicz, 2010) for discussions. The right‐hand sides of Equations 2 and 3 symbolize the forcing terms *R*_*x*_ and *R*_*y*_ for the momenta, such that

(5) $$R_{x}=-\frac{g}{h_{x}}\Phi \frac{\partial \left(\Phi +H_{0}\right)}{\partial x}+fQ_{y}+\frac{1}{G\Phi }\left(Q_{y}\frac{\partial h_{y}}{\partial x}-Q_{x}\frac{\partial h_{x}}{\partial y}\right)Q_{y}-\alpha \left(Q_{x}-{\bar{Q}}_{x}\right),$$

(6) $$R_{y}=-\frac{g}{h_{y}}\Phi \frac{\partial \left(\Phi +H_{0}\right)}{\partial y}-fQ_{x}-\frac{1}{G\Phi }\left(Q_{y}\frac{\partial h_{y}}{\partial x}-Q_{x}\frac{\partial h_{x}}{\partial y}\right)Q_{x}-\alpha \left(Q_{y}-{\bar{Q}}_{y}\right),$$

with specific terms, from left to right, representing the pressure gradient, the Coriolis force, the metric forces (viz. Christoffel symbols of the geospherical framework) and the relaxation forcings attenuating the solution to some hypothetical “true” state $\bar{Q}$ with the inverse time scale *α*. *H* = Φ + *H*_0_ with *H*_0_ denoting the topography, *g* is the gravitational acceleration and *f* the Coriolis parameter.

### 2.2 The Discretized Model

The below‐summarized procedure of formulating an implicit linear problem for dependent model variables, together with its associated Schur complement (i.e., elliptic Helmholtz equation) is a special case of a substantially more intricate procedure documented recently for all‐scale atmospheric Euler equations in (Smolarkiewicz et al., 2019), whereto the interested reader is referred for further details.

The governing Equations (1), (2), (3) are discretized in a SI fashion, using a collocated lat‐lon grid with its well‐known pole problem (Prusa, 2018). In a nutshell, momentum equations are integrated with the trapezoidal rule

(7) $$Q_{i}^{n+1}=M_{i}\left(Q^{n}+0.5\Delta tR^{n},v^{n+0.5},G\right)+0.5\Delta tR_{i}^{n+1}\;\equiv {\hat{Q}}_{i}+0.5\Delta tR_{i}^{n+1},$$

where *n* and **i** index discrete points (*t* ^*n*^, **x**_**i**_) separated with uniform intervals Δ*t* and (Δ*λ*, Δ*ϕ*), respectively, in time and the two spatial directions. The first term on the RHS of 7 denotes a second‐order MPDATA operator—with **v** ^*n*+0.5^ symbolizing an $O\left(\Delta t^{2}\right)$ explicit predictor of advective velocity at the intermediate *t* ^*n*^ + 0.5Δ*t* time level; cf. Sections 3.4 and 4.1 in Smolarkiewicz & Margolin (1998)—and it forms the explicit part of the solution. **R** ^*n*^ is readily calculated as it is a function of known variables. The second term however is unknown, as **R** ^*n*+1^ depends (nonlinearly) both on Φ^*n*+1^ and **Q** ^*n*+1^. There are several alternative approaches to assure an easily invertable linear problem, involving outer iterations or explicit predictors, or both (Kühnlein et al., 2019; Smolarkiewicz et al., 2016, 2017, 2019). As our goal is to assess the role of mixed precision on the elliptic solver performance, we here select an ad hoc approach that complicates (rather than simplifies) the target elliptic solver.

Specifically, the pressure gradient term is approximated as

(8) $${\left(\Phi \nabla H\right)}^{n+1}=0.5\left[\Phi^{n+1}\nabla \left(\Phi^{⋆}+H_{0}\right)+\Phi^{⋆}\nabla \left(\Phi^{n+1}+H_{0}\right)\right],$$

where Φ^⋆^ denotes the explicit second‐order‐accurate predictor of Φ^*n*+1^, generated by integrating the mass‐continuity Equation 1 with MPDATA. The Coriolis and relaxation terms are implicit at *t* ^*n*+1^, whereas the nonlinear metric forces—generally acting on a much longer time scale than the scale associated with the propagation of external gravity waves—are predicted explicitly at *n* + 1 by linear extrapolation from *n* and *n* − 1 time levels. The resulting integral of Equation (1), (2), (3) is by construction second‐order‐accurate in time and space; cf. (Kühnlein et al., 2019) for a pertinent discussion. Thanks to the collocated grid, the resulting linear problem is inverted analytically, forming the closed‐form expressions

(9) $$Q_{i}^{n+1}={\hat{\hat{Q}}}_{i}+A_{i}\nabla \Phi_{i}^{n+1}+B_{i}\Phi_{i}^{n+1},$$

where $\hat{\hat{Q}}$, **A** and **B** denote, respectively, the modified explicit part of the problem, and the 2 × 2 matrix and vector of known coefficient fields. Implementing Expression 9 in the trapezoidal integral of Equation 1,

(10) $$\Phi_{i}^{n+1}=\Phi_{i}^{n}+\frac{1}{2G_{i}}\left(\nabla_{i}\cdot Q^{n+1}+\nabla_{i}\cdot Q^{n}\right),$$

leads to the elliptic Helmholtz problem for Φ^*n*+1^, which can be symbolically written as

(11) $$\forall \;\left(n+1,i\right)\;\;L_{i}\left(\Phi^{n+1}\right)-R_{i}^{n+1}=0\;;$$

hereinafter, spatial and temporal grid indices are dropped as there is no ambiguity. The linear operator L is negative definite but not self‐adjoint. It takes the form of a generalized Laplacian

(12) $$L\left(\Phi \right)≔\sum_{I=1}^{M}\frac{\partial }{\partial x^{I}}\left(\sum_{J=1}^{M}A^{IJ}\frac{\partial \Phi }{\partial x^{J}}+B^{I}\Phi \right)-C\Phi ,$$

where the coefficient fields are straightforward modifications of those appearing in Equation 9, and *M* = 2. The coefficients for this shallow‐water model are given in Appendix A. The solution of Equation 11 provides the updated Φ—upon which **Q** ^*n*+1^ is recovered from Equation 9 and then **v** ^*n*+1^ from Equation 4—thus allowing to complete Equation 7 for calculations with large Δ*t*, limited only by the advective CFL condition.

### 2.3 The Elliptic Solver

**Figure 1:**
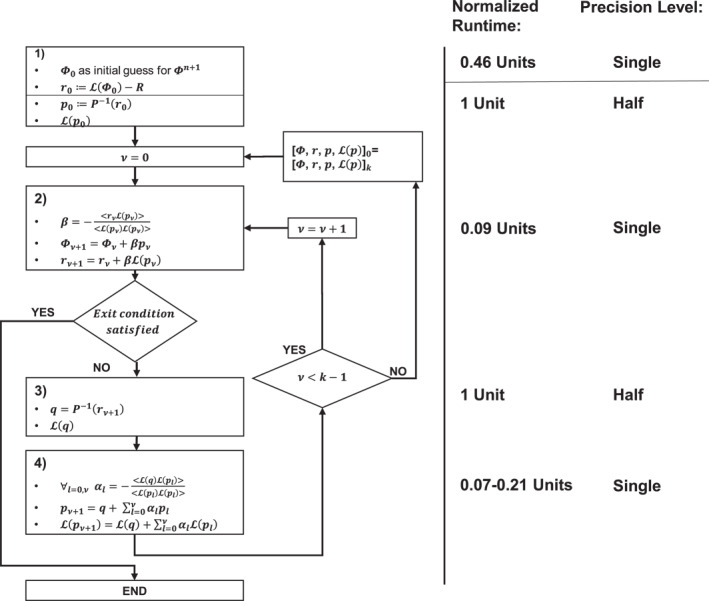
From left to right: a flow chart of our preconditioned GCR(k) implementation; measured runtimes (double precision) of the four distinct algorithmic steps, normalized by the runtime of algorithmic step 3, for GCR(3). These performance measurements have been performed on a single core (CPU: Intel i5, Fortran compiler: GCC 9.3.0.) and they agree with the ratio of the number of occurring floating point operations. We also provide the choice of base precision for each algorithmic step (the choices are described in more detail in Section 4.3).

The elliptic problem is solved using the preconditioned restarted generalized conjugate residual, GCR(k), approach (Eisenstat et al., 1983; Gillard & Benacchio, 2022; Smolarkiewicz & Margolin, 2000). GCR(k) is a Krylov sub‐space method that assures monotone convergence of the solver iterations for a non‐self‐adjoint operator L (i.e., ”non‐symmetric” in matrix representation) by minimizing 〈*rr*〉 (where 〈..〉 marks the domain integral), which is equivalent to the Euclidean norm *L*_2_(*r*) squared, of the residual error

(13) $$r_{\nu }=L\left(\Phi_{\nu }\right)-R,$$

here *ν* numbers the iterations. In essence, GCR(k) cleverly re‐optimizes at each iteration all coefficients in the series of the current and past successive iterations up to some arbitrary specified number, say *k* − 1; cf. (Smolarkiewicz & Szmelter, 2011) for a discussion. When after some number of iterations *N*, *L*_2_(*r*) reaches a desired small error tolerance, the solution of this iterative process Φ_*N*_ is set to be Φ^*n*+1^ ≔ Φ_*N*_. Figure 1 describes our implementation of the preconditioned GCR algorithm. Given the experience with the EULAG model (Prusa et al., 2008), GCR(3) is used.

Following the step 2 of the algorithm—the update of the depth Φ and the minimized residual error—there is an exit condition. While a variety of exit conditions can be considered (Smolarkiewicz et al., 1997), we found that demanding the uniform reduction of the initial residual error,

(14) $$\|r_{\nu +1}{\|}_{2}\;\leq \;\epsilon \|r_{0}{\|}_{2},$$

assures the stability of the SI model integrator while maintaining the solution quality throughout a range of spatial resolutions and physical scenarios. In practice, the choice of *ϵ* is verified experimentally. Based on our overall experience with W&C models, in this paper *ϵ* = 10^−5^ is assumed for all experiments. We also enforce at least one full GCR(3) cycle regardless of Equation 14. Further discussion and justification for this choice of *ϵ* is found in Section 4.1.

The preconditioner P in step 3 approximates L indirectly, by estimating the solution error $q=P^{-1}\left(r\right)\approx e=L^{-1}\left(r\right)$ by means of a stationary iteration—indexed with *μ*, such that *q*_*μ* = 0_ = 0—best characterized as a SI Richardson scheme (Smolarkiewicz & Margolin, 2000). Specifically, the operator P is split in two parts. The first part combines the second‐order zonal derivative term and the Helmholtz term, $P^{Z}-P^{H}$; whereas the second part, marked as $P^{M}$ for its meridional predominance, is the reminder of L and the first part. In the SI Richardson scheme, the first part is taken at the *μ* + 1 iteration while $P^{M}$ is lagged behind. This results in a tridiagonal problem

(15) $$\left[I-\eta \left(P^{Z}-P^{H}\right)\right]q_{\mu +1}=q_{\mu }+\eta \left[P^{M}q_{\mu }-r_{\nu +1}\right],$$

where I denotes the identity operator, and *η* can be interpreted as a pseudo‐timestep, determined from linear stability theory for the $P^{M}$ operator. For further reference and illustration, we implemented four preconditioning options in the SI shallow‐water model. Options 0 to 2 are elementary, in that: option 0 corresponds to P=I, viz. no preconditioning; option 1 is for P=D, where D symbolizes the diagonal part of L in 11; option 2 is a diagonally preconditioned explicit Richardson iteration for full L; and option 3 is the SI Richardson scheme in 15. Taking the number of GCR iterations with option 0 as a reference, the subsequent options reduce the solver's iteration count by about 10, 40 and 98 percent, respectively. In the experiments reported in this paper, we use solely option 3 with two Richardson iterations that comprise two tridiagonal inversions and one evaluation of $P^{M}$. We found that this combination yields the best overall performance.

## 3 Selected Test‐Cases and Experimental Setup

### 3.1 Preamble

To assess the impact of mixed precision on the performance of SI W&C models, we exploit two well‐established benchmarks for testing and evaluating shallow‐water surrogates of dynamical cores for global W&C, namely the Rossby‐Haurwitz wave with wavenumber four (RHW4) and a zonal flow (*Q*_*x*_/Φ ∝ cos *ϕ*, *Q*_*y*_ = 0) past the Earth orography. The Rossby‐Haurwitz waves are analytic solutions of the nondivergent nonlinear barotropic vorticity equation on the sphere, and as such propagate zonally without change of shape (Haurwitz, 1940). However, Rossby‐Haurwitz waves are incompatible (Temam, 2006) with SWE, and their SWE simulations lead to unstable solutions, given sufficiently short zonal wavelength and sufficient amplitude (Hoskins, 1973). In particular when simulated with SWE, the form of RHW4 changes a little over 24 days, while twice shorter Rossby‐Haurwitz waves break completely within a week (Hoskins, 1973). Because of this marginal stability (Thuburn & Li, 2000), RHW4 is a convenient vehicle to test capability of numerical methods for maintaining subtle nonlinear balance of wave form solutions over an extended integration time. The RHW4 simulation with SWE was included in the suite of tests proposed by Williamson et al. (1992), and has become a prominent benchmark in the field.

As the RHW4 problem is particularly smooth, with the analytic solution confined to a few spherical harmonics (Thuburn & Li, 2000), we complement it with a multi‐scale variant of the classical problem of a geostrophically balanced zonal flow past a hill centered in mid latitudes. The original problem has been studied in (Grose & Hoskins, 1979). It is characterized by small Rossby and Froude numbers—here *R*_*o*_ = *U*/*Lf* and $Fr=U/\sqrt{gH}$, with *U*, *L* and *H* denoting the characteristic flow speed, horizontal scale of the hill and the mean height of the shallow‐water surface, respectively—whereby it is well explained by the linear theory (Grose & Hoskins, 1979). This problem, also proposed by Williamson et al. (1992) for evaluating the efficacy of numerical methods for global‐scale dynamics, has become a benchmark in the field—see for example, (Smolarkiewicz et al., 2001) for its 3D nonhydrostatic extension. Here we retain the benchmark setup as proposed in (Williamson et al., 1992), except for replacing the smooth localized hill with Earth's orography. This effects in numerical solutions with broad spectra of scales and, effectively, a stiffer elliptic problem.

To quantify the impact of the reduced precision on the solver convergence and solutions quality, we adapt the *L*_2_ (Euclidean) and *L*_*∞*_ (infinity) error norms defined in (Williamson et al., 1992) as normalized deviations from a “genuine” solution. For the RHW4 test‐case, the genuine solution is the analytic solution $\Psi_{0}\left(x-Vt,y\right)$, where **Ψ**_0_ = **Ψ**(*x*, *y*) represents the initial condition for the velocity and depth fields—given in eqs. (143), (144), and (146) of (Williamson et al., 1992)—and V is the propagation speed of the analytic Rossby‐Haurwitz wave (eq. 142 in (Williamson et al., 1992)). As the analytic wave does not satisfy the shallow‐water Equations (1), (2), (3), the deviations of numerical solutions from $\Psi_{0}\left(x-Vt,y\right)$ are expected to grow in time. However, the comparison provides a meaningful gauge for assessing the impact of a reduction in precision. In the orographic flow test‐case the genuine solution is specified as a geostrophically balanced zonal flow, steady in the absence of the orography. For both benchmarks, the calculations addressing reduced precisions are conducted on the three regular longitude‐latitude grids, with respective resolutions *NX* × *NY* = 128 × 64, 256 × 128, and 512 × 256.

### 3.2 Highlights of RHW4 Simulations

**Figure 2:**
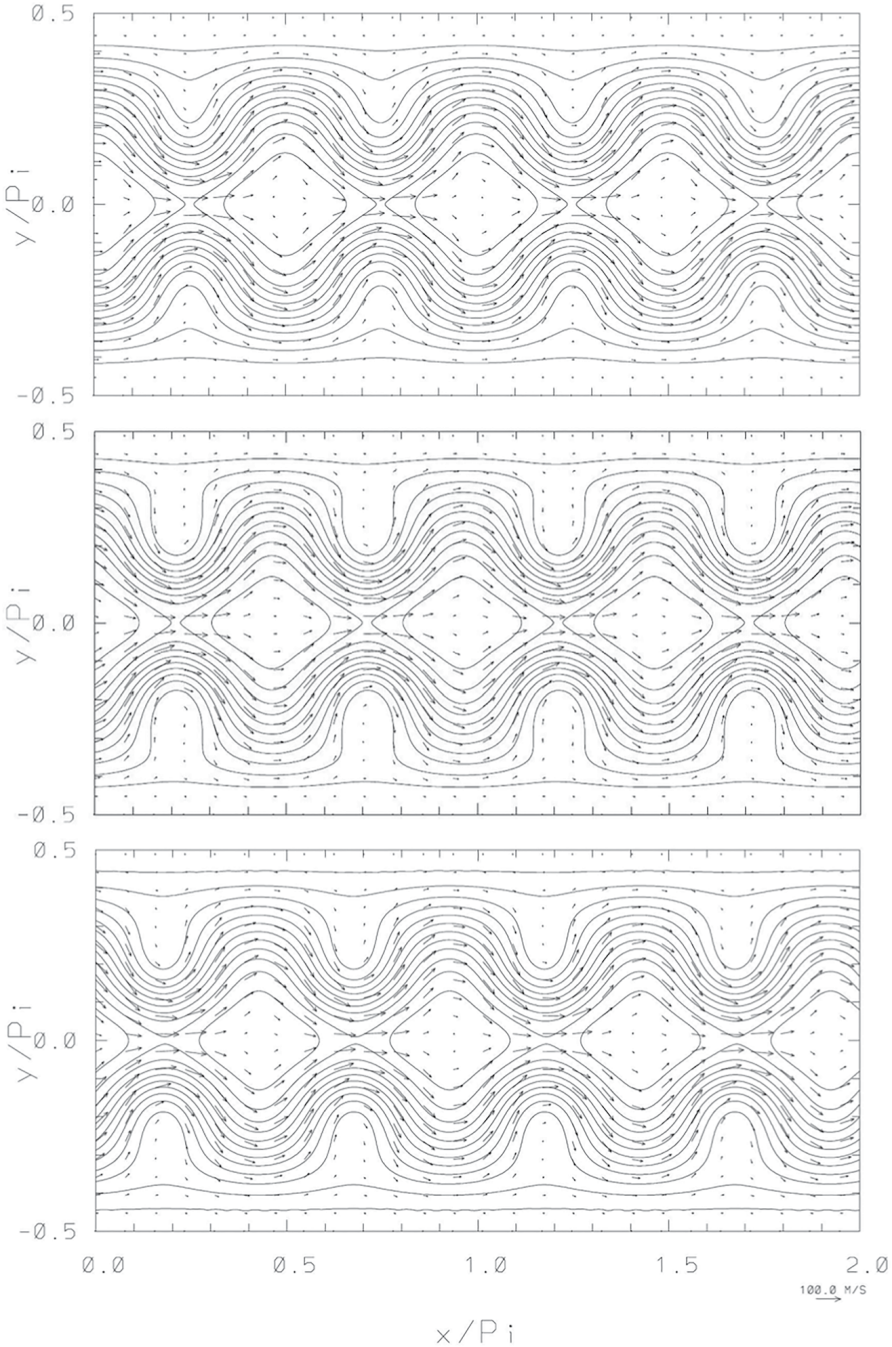
RHW4, instantaneous normalized free‐surface perturbations (Φ(*x*, *y*, *t*) − *H*_*o*_)/*H*_*o*_ (*H*_*o*_ = 8 km), with imposed corresponding flow vectors. From the top to bottom, respectively, are the initial condition and the numerical results after 7.38 and 14.76 days, corresponding to one and two periods after which the analytic RHW4 propagates by one and two full wavelengths. The perturbation isolines are plotted with the contour interval 0.025 (zero contour lines are not shown), and the reference 100 m/s flow arrow is shown in the lowest right corner.

**Figure 3:**
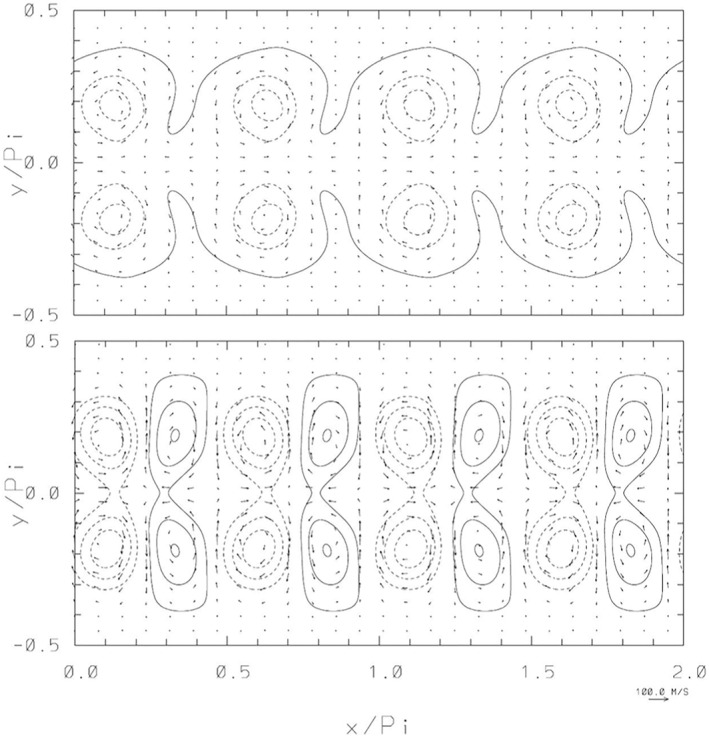
RHW4, the normalized free‐surface departure from the analytic result, $\left(\Phi \left(x,y,t\right)-\Phi_{0}\left(x-Vt,y\right)\right)/H_{o}$, with imposed corresponding departures of the flow vectors. The top and bottom panels show the results after *t* = 7.38 and *t* = 14.76 days, respectively. The contour interval and the reference flow arrow are the same as in Figure 2; zero contour lines are not shown and the dashed contours correspond to negative values.

Figure 2 shows the analytic initial condition and the numerical results on the 512 × 256 grid after one and two periods of the analytic RHW4 when the analytic solution exactly reproduces the initial condition. The departures from the analytic result are clearly seen. After one period, the numerical RHW4 is steeper and somewhat retarded compared to the analytic result. After the two periods, the numerical results is less steep but the phase retardation is more pronounced. The lower resolution results (not shown) look similar, which is not surprising as the RHW4 is well resolved (with 32 grid intervals per wavelength) even on the coarsest grid; cf. Figure 2 in Smolarkiewicz & Margolin (1998) that shows a coarse result after 5 days. As expected (see Figure 3 in Thuburn & Li (2000)), the current solution is visibly stable, which however should not be taken for granted, as irregularities of model discretization can excite instability of the RHW4 already after 5 days; cf. Figures 11 and 12 in Szmelter & Smolarkiewicz (2010). Figure 3 complements Figure 2 with the display of relative departures of the numerical solutions from the analytic results.

To appreciate the challenging nature of the illustrated calculations, consider that the gravity wave speed ($\sqrt{gH_{o}}=280$ m/s) is comparable to the speed of sound, while the zonal length interval of the physical grid in the rings of grid points adjacent to the poles is *δx* = 7,675, 1,919 and 480 m for the three considered regular grids. The explicit solutions, like those discussed in (Smolarkiewicz & Margolin, 1998; Szmelter & Smolarkiewicz, 2010), require then temporal intervals Δ*t* ∼ 40, 10, and 2.5 s, respectively, whereas SI calculations reported here were conducted with Δ*t* = 800, 400, and 200 s, respectively. The attained linear rather than quadratic reduction of Δ*t* with the reciprocal of *NY* is due to the zonal flow decaying toward the poles. Although the calculations employed weak polar absorbers—with the inverse time scale *α* increasing linearly from zero at the angular distance *ϕ* ≥ 3*π*/64 away from the poles to 1/(200Δ*t*) at the poles—essentially the same solution can be obtained without any absorbers. However, even weak absorbers can improve the solver convergence by improving diagonal dominance of the linear operator (Equation 12). For example, the presented results using weak polar absorbers *α* = 1/(200Δ*t*) have a 30% reduced Euclidean norm of the residual ‖*r*_*N*_‖_2_ on average when exiting the GCR algorithm (on average 4 iterations) compared to using no polar absorbers (*α* = 0 everywhere). The calculations illustrated in Figures 2 and 3, evinced the maximal Courant numbers 118 and 0.31, correspondingly with respect to the gravity wave speed and the flow speed.

### 3.3 Highlights of Orographic Flow Simulations

**Figure 4:**
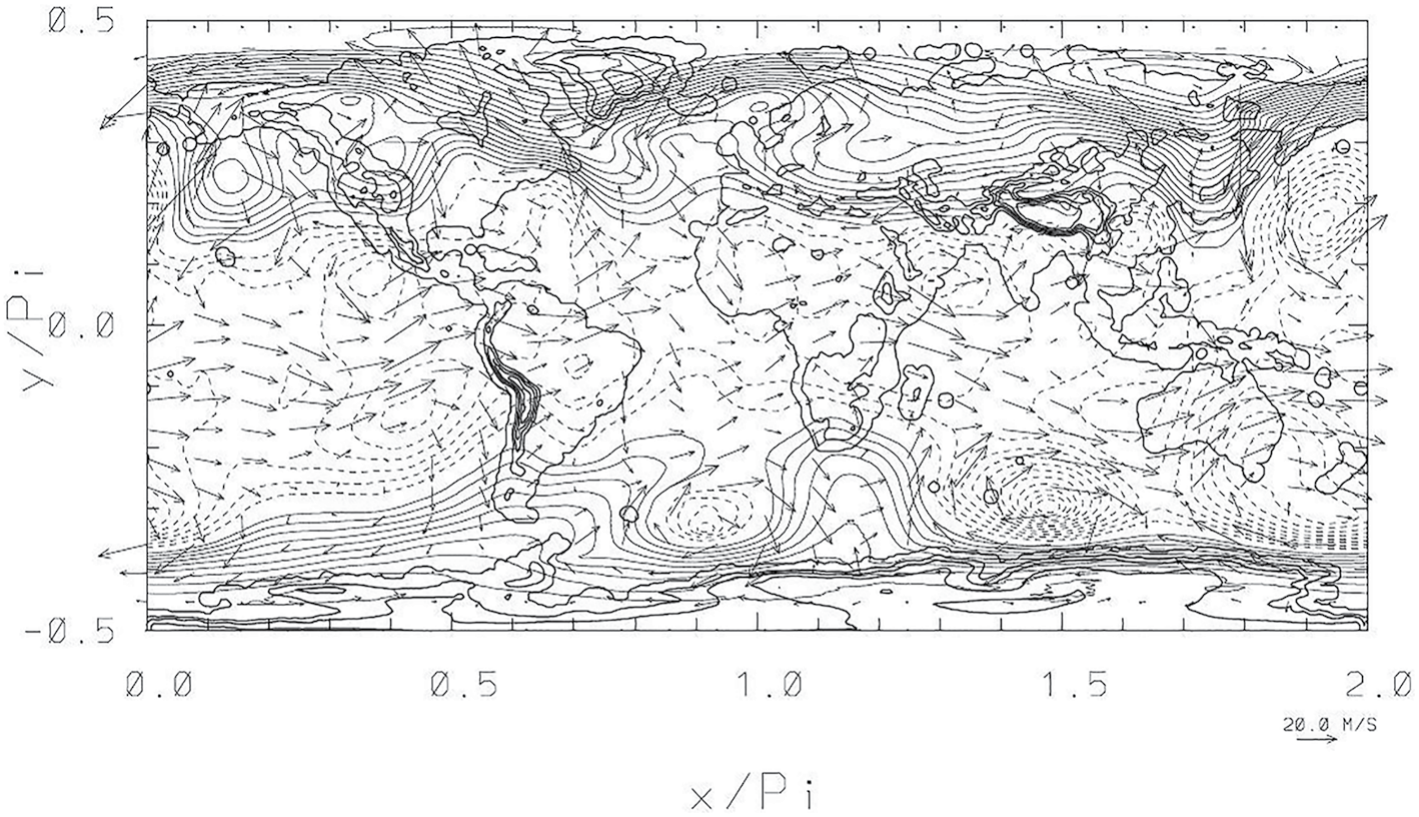
Normalized free‐surface height perturbations (*H*(*x*, *y*, *t*) − *H*(*x*, *y*, 0))/*H*_*o*_ (here *H* = Φ + *H*_0_ and *H*_*o*_ = 8 km), with imposed corresponding flow vectors at *t* = 14.76 days. The perturbation isolines are plotted with the contour interval 0.00625 (dashed lines are for negative values and zero contour lines are not shown), and the reference 20 m/s flow arrow is shown in the lowest right corner; the contours of the orography *H*_0_(*x*, *y*) span the range 3–7,003 m with the 1,200 m interval.

In analogy to the RHW4 case, Figure 4 shows the numerical results for the orographic flow simulation on the 512 × 256 grid after 14.76 days, likewise simulated with Δ*t* = 200 s. Pattern‐wise, the departures from the initial zonal flows are dramatic, especially in terms of energy and direction of local flows. Quantitatively, the free‐surface height perturbations (viz. pressure perturbations) are about 3% and 12% in terms of *L*_2_ and *L*_*∞*_ norms; that is, consistent with real weather (Smolarkiewicz et al., 2019). For reference, the same measures for the RHW4 simulation are of comparable size, with 3% and 7% respectively. In terms of the kinetic energy of the velocity perturbations, the corresponding *L*_2_ and *L*_*∞*_ norms are 153% and 762%, with the latter reflecting both the direction (say easterly or northerly) and the high speed of local flows (∼ 50 m/s) emerging in the high latitudes of the northern hemisphere. The respective values for the RHW4 case are much smaller in comparison, with 37% and 35%. The maximal Courant numbers with respect to the gravity wave speed is 116, which is about the same as in the RHW4 case. The maximal Courant numbers with respect to flow velocity is 0.71, which is more than doubled compared to the RHW4 case. Nonetheless, the overall performance of the elliptic solver was comparable at 4 GCR iterations per time step. The essential difference between the orographic flow simulation and the RHW4 setups is in the importance of the absorbers, its inverse time scale here taken as *α* = 1/(2Δ*t*) consistently with the theoretical considerations (Prusa, 2018).

### 3.4 Reduced‐Precision Experiments

The precision reduction is achieved in three distinct steps. In the first step, precision is reduced from double to single precision (23 significant bits) for the entire shallow‐water model. Unfortunately, solver convergence is compromised if single precision is used for the entire model. The results of these experiments are presented in Section 4.1. Based on the experience from the tests with the single precision shallow‐water model, a mixed‐precision shallow‐water model is described in Section 4.2, that behaves similarly to the DP reference model. As single precision arithmetic is easily available on modern CPUs, these reduced‐precision experiments can be performed on standard hardware. In the third step, a mixed‐precision model is presented in Section 4.3 that is additionally using half precision for most parts of the elliptic solver. Half precision is emulated with the reduced‐precision emulator (rpe v5 library (Dawson & Düben, 2017)). Using the emulator enables us to explore a precision reduction beyond single precision with model code that was developed for standard computer hardware.

While we emulate the 10 significant bits as in half precision, we keep the number of exponent bits at 11—the DP standard—which means that the dynamical range of representable numbers remains high. The results should still be representative for the use of half precision as problems with the dynamical range can often be mitigated via rescaling of variables, see for instance (Klöwer et al., 2019), especially since the elliptic problem is linear by design. In Section 4.4, the topic of rescaling is again picked up when performance is measured on actual half precision hardware.

The precision levels for the mixed‐precision elliptic solver are identified as the minimum precision required for each step of the algorithm shown in Figure 1. Because the elliptic solver is part of the timestepping scheme of a non‐linear model, it is not obvious how this could be achieved via analytical means. Thus, we adopt an experimental approach with the precision being reduced to levels where the elliptic solver still yields acceptable results. The elliptic solver's performance is studied for each of the two test‐cases defined earlier. This covers a wide range of different dynamics, from linear responses up to turbulent regimes with a multiplicity of scales.

We consider three criteria to measure the performance and viability of the elliptic solver when precision is reduced: (i) the solver’s convergence rate; (ii) the solver accuracy (14); and (iii) the deviation of the model solution from the respective “genuine” solution in the *L*_2_ and *L*_*∞*_ error norms. Criterion (i) aims at keeping the solver's convergence rate unchanged compared to the reference DP solver. A decrease in the solver's convergence rate, and thus an increase in the expected solver iterations, would be acceptable if each iteration is sufficiently cheap when using reduced precision. However, we would argue that it is still reasonable to aim for approximately the same number of solver iterations in the mixed‐precision solver, especially if one aims for optimal or near‐optimal speed‐ups close to the theoretical limit. Criterion (ii) is straightforward. As soon as the precision reduction is introduced into parts of the solver, there is a risk that the residual errors become corrupted with rounding errors. It is therefore important to show that the elliptic solver is actually capable of converging to the required accuracy. Criterion (iii) is more subtle. The solver accuracy is chosen based on the additional error in the model solution of a DP reference run, defined as the relative error between the accuracy measures with respect to genuine solutions specified in the last paragraph of Section 3.1. Given that the residual‐error fields of a DP and a mixed‐precision elliptic solver will be different—e.g., due to a different spectral composition—even if the required solver accuracy was reached, the pattern and magnitude of the additional discretization error might change as well.

## 4 Results

In this paper, the focus is primarily on the 512 × 256 resolution, the highest of the three resolutions, as it shows the strongest grid convergence at the poles and is thus expected to be the most challenging test‐case for reduced precision.

To evaluate the solver's convergence rate and the solver accuracy, the discussed residual error fields are calculated by using Definition 13, in DP arithmetic, applied to the updated fluid thickness field Φ_*ν*_. We do not use the minimized residual error field *r*_*ν*_ from step 2 of the GCR algorithm in Figure 1 as the values of *r*_*ν*_ may already be corrupted by rounding errors.

Given these choices, the convergence plots that are an integral part of the following discussion are here introduced in detail to avoid ambiguity. In the convergence plots, the development of the residual error field in the Euclidean norm, as defined in the exit condition 14, is shown over successive solver iterations. We number the solver iterations as follows: iteration 0 denotes the initial residual error field $L\left(\Phi_{0}\right)-R$, iteration 1–3 denote the residual error fields $L\left(\Phi_{\nu +1}\right)-R,\;\nu =0,1,2$ of the first cycle of the GCR(3) algorithm. Following this convention, iteration 4–6 denote the fields $L\left(\Phi_{\nu +1}\right)-R,\;\nu =0,1,2$ of the second cycle of the GCR(3) algorithm. To highlight the residual error field's evolution over successive solver iterations, values stemming from different iterations of the same call to the elliptic solver are connected by lines.

### 4.1 Single Precision Shallow‐Water Model

**Figure 5:**
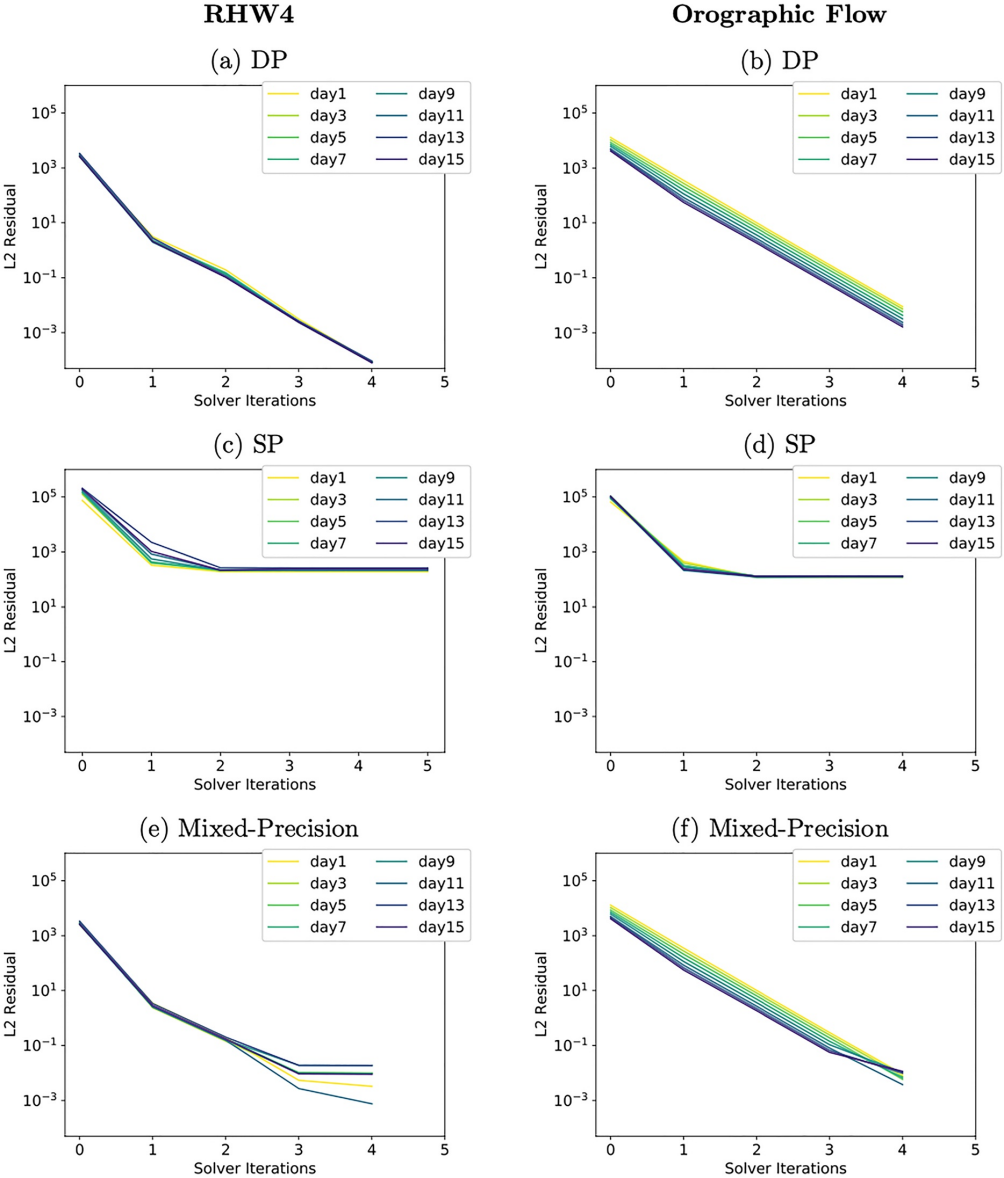
Convergence rates of the RHW4 (left) and the orographic flow test‐case (right) for three variants of the shallow‐water model, (a and b) use double precision arithmetic, (c and d) single precision, (e and f) a mixture of double and single precision. Convergence is shown for 2‐day time interval snapshots, and darker colors indicate later simulation time.

In DP arithmetic, the solver is converging steadily throughout all solver iterations, see Figures 5a and 5b. The solver requires 4 solver iterations for both test‐cases which is also the minimal iteration number since we enforce for at least one full cycle of GCR(3). In 3D atmospheric models GCR(3) requires on average between 4 and 40 iterations per time step, depending on the complexity of the simulated flow, the equations solved, the used preconditioner, the stopping criteria and the grid resolution (Prusa et al., 2001; Smolarkiewicz et al., 1997).

Statistics of the increments which are used to update the fluid thickness after each timestep are shown in Table 1. For both test‐cases, fluid thickness increments drastically reduce in magnitude over successive solver iterations by 5–6 orders of magnitudes. For the RHW4, this process happens more quickly, consistent with the slightly higher convergence rate for this test‐case.

**Table 1:** Size of the Increments for Fluid Thickness Φ_0_ for the RHW4 and the Orographic Flow Test‐Case at Different Iterations of the Elliptic Solver for the Double Precision Shallow‐Water Model

Solver iteration	1	2	3	4
RHW4	0.6 m	5 ⋅ 10^−4^ m	2 ⋅ 10^−5^ m	5 ⋅ 10^−7^ m
	3.5 m	2 ⋅ 10^−3^ m	3 ⋅ 10^−4^ m	6 ⋅ 10^−6^ m
Orographic Flow	1.8 m	4 ⋅ 10^−2^ m	1 ⋅ 10^−3^ m	3 ⋅ 10^−5^ m
	48 m	1.1 m	4 ⋅ 10^−2^ m	8 ⋅ 10^−4^ m

*Note.* The upper values provide the average magnitude and the lower values the maximum value throughout the model run.

Given these results, the significance of using *ϵ* = 10^−5^ should be discussed. For the orographic flow test‐case, the fluid thickness increments can still be up to several centimeters in magnitude until the fourth solver iteration. Errors on a magnitude of centimeters within a single time‐step are comparatively large if put into perspective with Table 1 which shows that the *L*_2_‐norm of the entire solver increment in Φ is on the order of only 2 m itself. For the RHW4, the strongly decreased fluid thickness increments after the first solver iteration suggest that it would be sufficient to only perform one single solver iteration, or at most two. However, DP shallow‐water model experiments with an elliptic solver restricted to only one or two solver iterations respectively lead to model crashes due to exponentially growing instabilities, in the case of a single solver iteration even within the first 12 hr of simulation time.

In comparison to the DP convergence behavior, single precision solver convergence rates seemingly stall after the first solver iteration, see Figures 5c and 5d.

While there are differences in the convergence, concerning criterion (iii)—the deviations from the genuine solution—the solution quality is still good. The differences in the *L*_2_ and *L*_*∞*_ norms compared to the DP solution are insignificant. In this, the deviations of a model solution are defined at the example of DP for the kinetic energy as follows. It is the kinetic energy of the velocity field differences between the DP solution and its “genuine” solution: **Q**_**DP**_/Φ_*DP*_ − **Q**_**0**_/Φ_0_. The largest difference between double and single precision is found for the *L*_*∞*_‐norm of kinetic energy deviations for the orographic flow with a value of 758% for the single precision shallow‐water model, a relative change of 5 ⋅ 10^−3^ compared to the DP solution.

**Figure 6:**
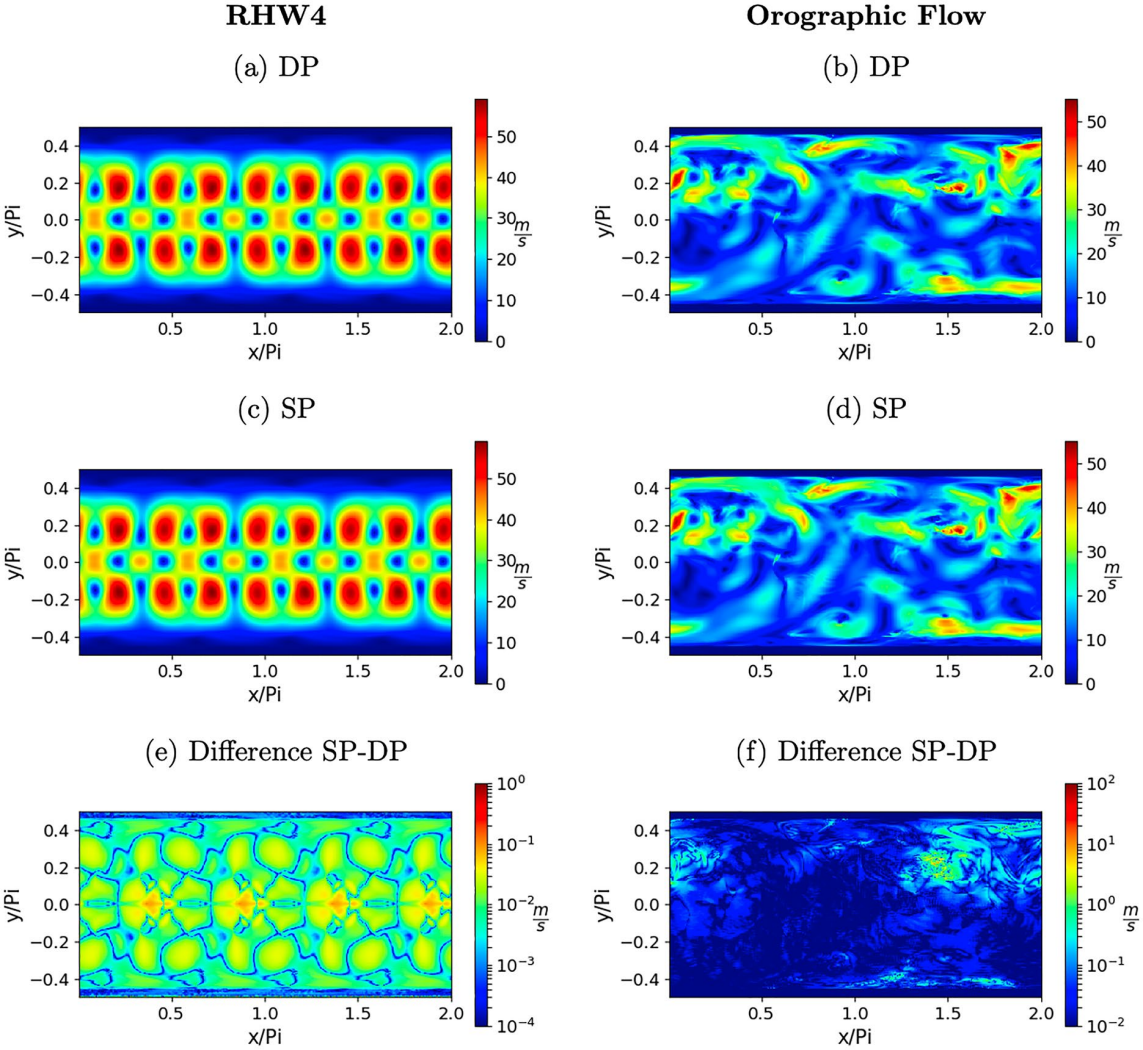
Square‐root of kinetic energy deviations in $\frac{m}{s}$ from the respective “genuine” model solution at *t* = 14.76 days for the RHW4 in (a) double precision, (c) single precision, and the orographic flow test‐case in (b) double precision, (d) single precision. Figures (e and f) show the respective difference in kinetic energy deviations between the double precision and single precision shallow‐water model solution in logarithmic scale.

Further elaborating on this point, the above‐discussed kinetic energy deviations as well as their change when going from double to single precision are shown in Figures 6a–6f. The differences shown in Figures 6e and 6f are the absolute values of the differences between Figures 6a and 6c and 6cand6d, respectively. To be more comparable with the figures showing the model solutions itself, here the square‐root of kinetic energy is shown. For both test‐cases and both precision levels the deviations in kinetic energy can be up to 50 m/*s* for both test‐cases.

For RHW4 the deviations from the genuine solution are centered around the wave crests and symmetric between both hemispheres. The absolute values of the differences between the DP perturbations and the single precision perturbations is shown in Figure 6e). The differences are at least two orders of magnitude smaller than the values of the deviations.

For the orographic flow, the deviations from the genuine solution show the locations and magnitude of synoptic‐scale eddies, as well as regions with high mountains, such as Himalaya, where fluid thickness is small compared to orography and orography gradients are large making the associated flow exhibit strongly non‐linear behavior. The differences, see Figure 6f), are about one order of magnitude smaller than the deviations themselves.

Differences in model solution between double and single precision need to be further put into perspective. Simulations of atmosphere and ocean models suffer from a number of errors that influence results, including imperfect initial or boundary conditions, discretization errors, or unknown physics for some of the model components. It is therefore important to put the impact of rounding errors into the context of other sources of errors. If the impact is small, a reduction in precision will likely have no measurable impact on prediction quality. This approach has been applied in a number of publications already (Düben & Dolaptchiev, 2015; Düben et al., 2014, 2017; Hatfield et al., 2018). Here, we add a random perturbation (white noise with an amplitude of five cm) to the initial conditions of the DP shallow‐water model of which we know that they will be within the level of initial condition uncertainty. It is found that the differences in kinetic energy deviations between the perturbed and unperturbed DP simulations are comparable to the differences between the simulations in single and DP. Thus, the change in deviations when going to single precision will probably be insignificant when compared to the uncertainty range typically encountered in W&C prediction.

In summary, although the solver of the single precision model variant behaves differently, the model still produces valid solutions for our test‐cases.

To better understand the convergence behavior of the single precision shallow‐water model, first, the potential impact of the absorbers is studied. However it is found that they behave exactly the same for the double and the single precision shallow‐water model. The RHW4 test‐case still works even without a polar absorber, while the orographic flow test‐case crashes within the first 24 hr of integration time regardless of whether single precision or DP is used in the shallow‐water model.

The convergence behavior of the single precision model is further investigated by looking at convergence in different locations of the computational domain. For this, the computational domain is split into a pole region (10 zonal bands wide in the vicinity of each pole) and the remaining computational domain which we here refer to as the interior. For the pole region, convergence looks exactly like the overall convergence shown in Figures 5c and 5d. For the interior of the domain, the first observation is that there is no increase in the initial residuals *r*_0_ when compared to the DP shallow‐water model. This shows that the observed increased values of the initial residual *r*_0_ stem from the pole region exclusively. Concerning convergence in the interior region, three solver iterations are required to reduce the initial residual field *r*_0_ by 3 orders of magnitude, showing the importance of performing more than one solver iteration. In contrast to the single precision solver, in the DP shallow‐water model, convergence of the first and its subsequent solver iterations is consistent everywhere, regardless of whether it is for the pole region or the interior. These descriptions are valid for both test‐cases.

To further understand why the single and DP solvers show different convergence behaviors, we take a look at the fluid thickness variable *Φ*. The fluid thickness variable is stored in single precision arithmetic and its magnitude is on the order of 10^4^–10^5^ m. In comparison, a typical fluid thickness increment at each time‐step is on the order of (see again Table 1) 3 ⋅ 10^−5^ to 5 ⋅ 10^−7^ m for the last solver iteration. However, the relative error of single precision truncation is given by the machine epsilon ≈1.19 ⋅ 10^−7^, indicating that thickness increments smaller than 10^−2^ to 10^−3^ m cannot reliably be represented in the fluid thickness field Φ.

**Figure 7:**
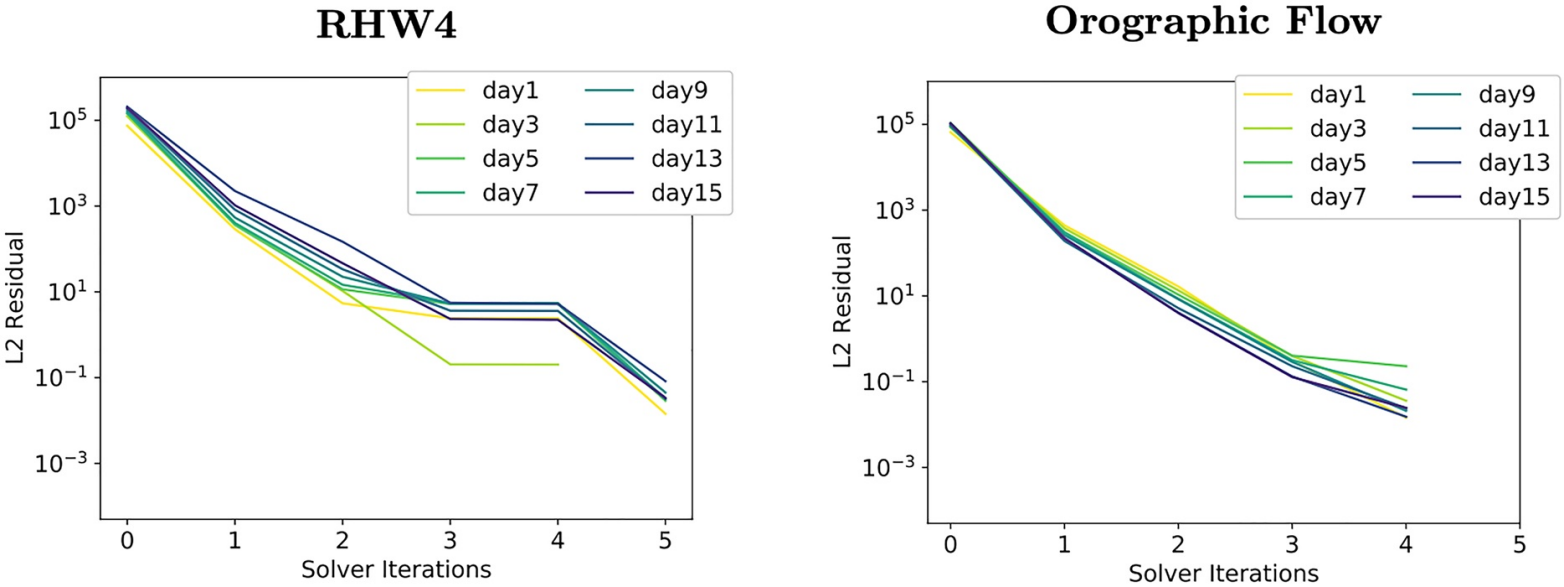
Convergence rates of the RHW4 (left) and the orographic flow test‐case (right) for the shallow‐water model using single precision. In contrast to Figures 5c and 5d, the residual error fields are however calculated from a double precision copy of the initial fluid thickness Φ_0_ i.e., updated by the solver increments after each solver iteration.

To study the effect of the increments that are too small to be represented in single precision, a DP copy of the initial fluid thickness Φ_0_ is introduced into the single precision elliptic solver and the residual error fields are calculated from this DP variable. The DP copy of Φ_0_ is continuously kept updated by the single precision solver's fluid thickness increments after each solver iteration. The convergence rates derived in this way, see Figure 7, look drastically different and much closer to the convergence rates of the DP variant of the elliptic solver from Figures 5a and 5b. The initial residual is still larger when compared to the DP simulations, but the residual error is now reduced at a comparable rate.

**Figure 8:**
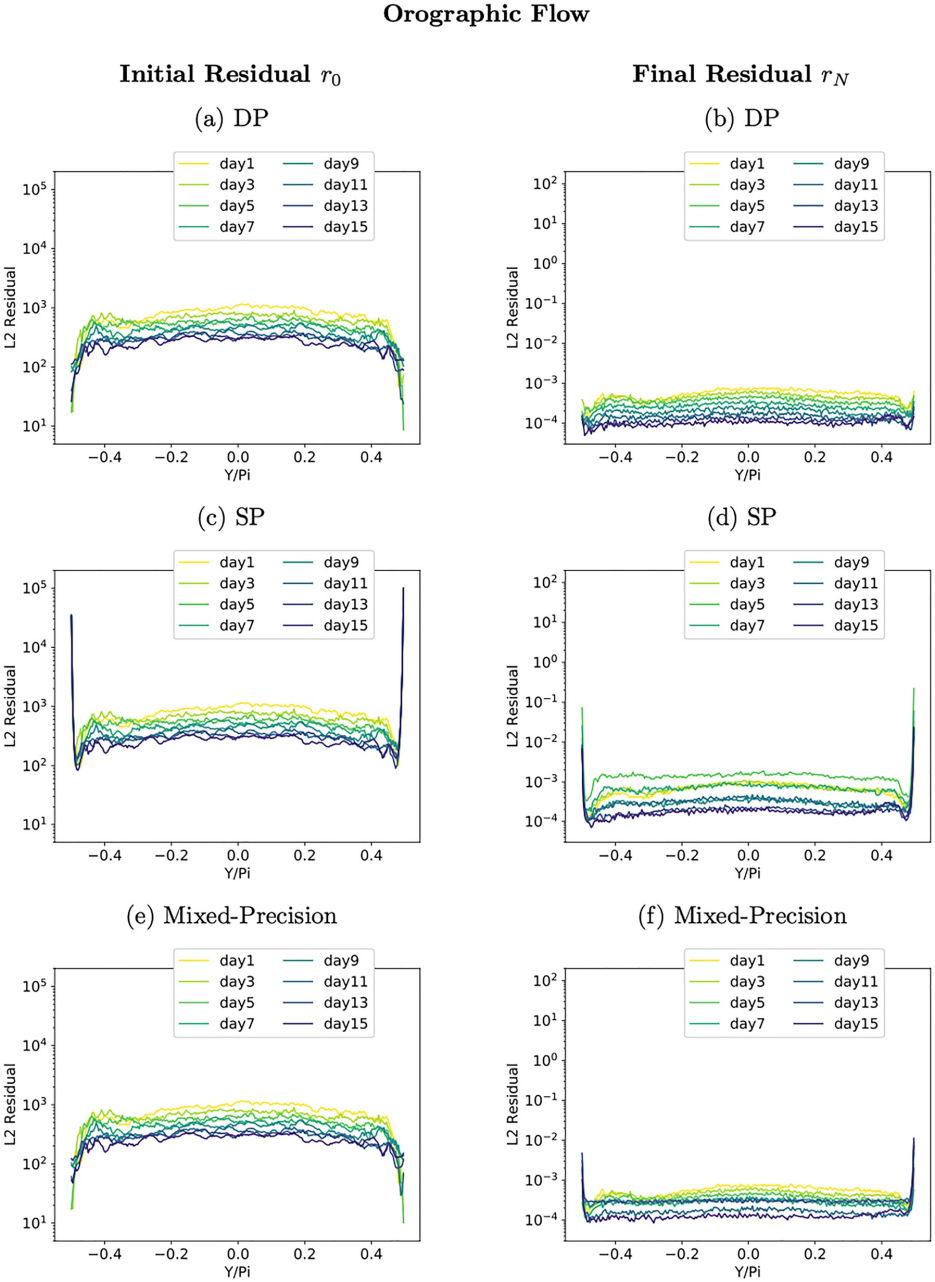
Latitude‐wise initial and final residual in the *L*_2_‐norm for the orographic flow test‐case for three variants of the shallow‐water model, (a and b) use double precision arithmetic, (c and d) single precision, (e and f) a mixture of double and single precision.

In the following, the solver's convergence is investigated for each latitude separately. For this analysis, the residual error fields are calculated from a DP copy of Φ. Figures 8a and 8d show the convergence rates for each latitude. Here only results for the orographic flow are shown as the RHW4 test‐case behaves qualitatively similar. The differences in the *L*_2_‐norm of the residual values are only visible near the pole. For the other latitudes, the *L*_2_‐norms of the residual error fields are indistinguishable by eye for single and DP.

**Figure 9:**
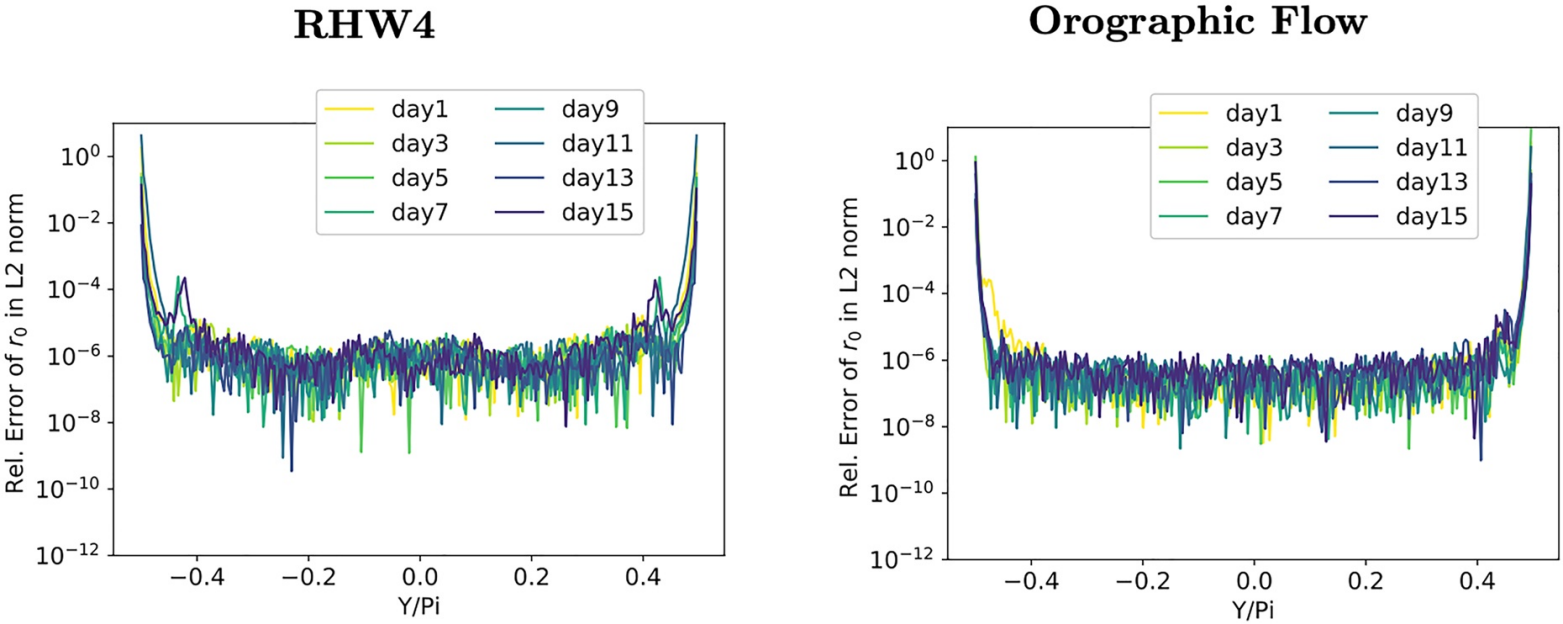
Latitude‐wise relative numerical error in the *L*_2_‐norm of the initial residual *r*_0_ when the elliptic solver's initial residual is calculated with a fluid thickness variable Φ_0_ that was truncated to single precision. Results are shown for the RHW4 (left) and the orographic flow test‐case (right) in 2‐day time interval snapshots; darker colors indicate later simulation time.

**Figure 10:**
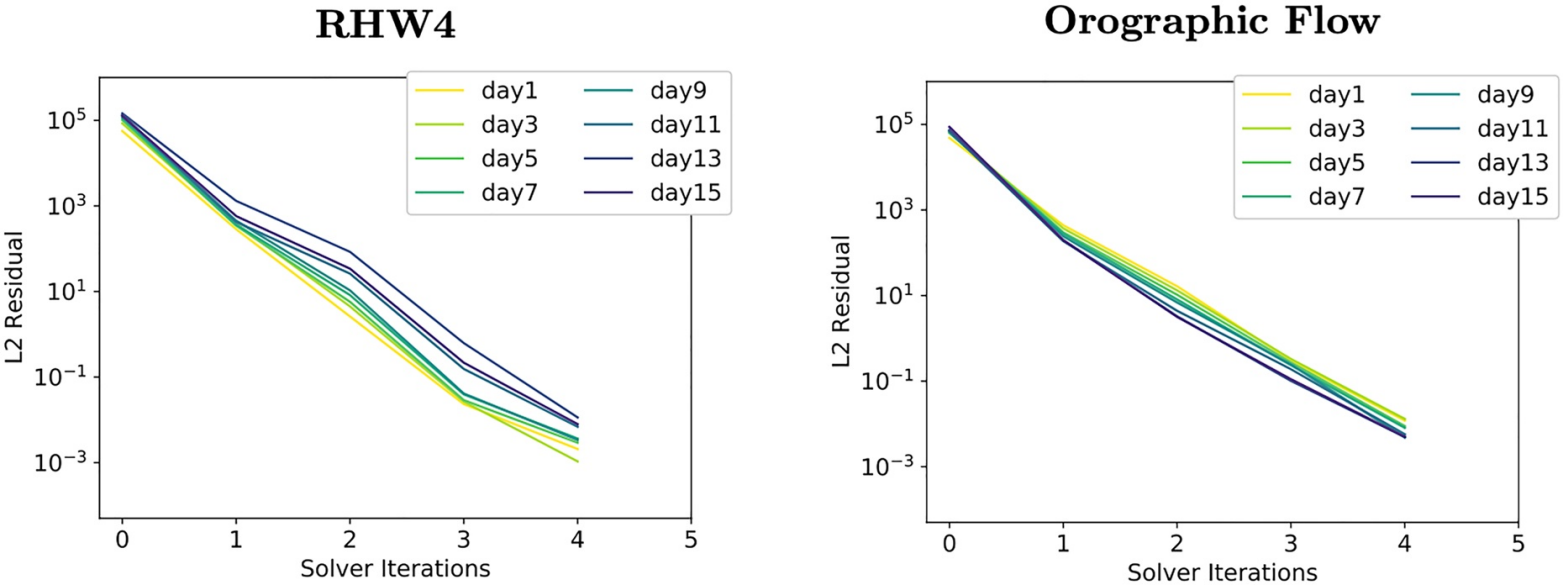
Convergence rates of the RHW4 (left) and the orographic flow test‐case (right) for the double precision shallow‐water model variant where, in contrast to Figures 5a and 5b, the elliptic solver's initial residual *r*_0_ is calculated with a fluid thickness variable Φ_0_ that was truncated to single precision.

The accumulation of numerical errors near the poles of the single precision model can be replicated in the DP model variant when the elliptic solver's initial residual *r*_0_ is calculated with a fluid thickness Φ_0_ that was truncated to single precision—even if all model calculations are performed in DP arithmetic. The spatial distribution of the numerical errors is illustrated in Figure 9. The relative error in the *L*_2_‐norm is around 10^−5^ for the majority of latitudes for both test‐cases, while the relative numerical errors in the *L*_2_‐norm at the poles can easily be as large as 100%. That a small perturbation on the order of the single precision epsilon results in relative errors of this magnitude after applying L, shows how badly conditioned the linear operator L is near the poles. The resulting solver convergence of the truncated elliptic solver is illustrated in Figure 10, showing a clear degradation when compared to the DP elliptic solver from Figures 5a and 5b.

The precision of the fluid thickness variable Φ_0_ is thus of major importance for the calculation of the initial residual error field *r*_0_ as well as the subsequent convergence behavior of the elliptic solver. For substantiation, additional experiments are run using stronger polar absorbers. For the RHW4, the single precision model variant is run with *α* = 1/(2Δ*t*) but it is found that, although the numerical errors in the *L*_2_‐norm of the initial residual is reduced by half, solver convergence is not improved.

### 4.2 Mixed‐Precision Shallow‐Water Model

Here, a mixed‐precision model is described that shows a very similar convergence behavior when compared to the DP variant while performing as many calculations as possible in single precision.

First, based on our previous analysis, the fluid thickness field Φ used for the initial residual *r*_0_ calculation in algorithmic step 1 of the elliptic solver and the update of the model variable Φ in step 2 should be kept in DP. This motivates an approach to keep the prognostic model variables Φ, *Q*_*x*_, and *Q*_*y*_ in DP while the costly part, the calculation of their respective tendencies within a time‐step, are calculated in single precision arithmetic. Updating the fluid thickness field Φ_*ν*_ directly in algorithmic step 2 is replaced by accumulating the solver increments to fluid thickness in a single precision variable ΔΦ_*ν*_ which is then added to the fluid thickness field Φ^*n*^ when the elliptic solver is exited. Second, the initial residual *r*_0_ calculation is performed in DP to ensure that the relative error in *r*_0_ is smaller than *ϵ*. And third, we find that the forces ${R_{i}}^{n}$—the forcing terms going into the second‐order MPDATA operator in Equation 7—need to be calculated in DP to avoid numerical errors at the poles. Again, the fluid thickness field Φ plays a crucial role as, similarly to the calculation of *r*_0_, the pressure gradient term from ${R_{i}}^{n}$ is responsible for a large fraction of the numerical errors at the poles. Although calculated in DP, ${R_{i}}^{n}$ is then only stored in single precison for all subsequent calculations.

The majority of the model is still performed in single precision. This includes the entire second‐order MPDATA operator (first term on the RHS of Equation 7), and the calculation of the advective velocities **v** ^*n*+0.5^.

These precision choices already define most of the mixed‐precision shallow‐water model except for the precision choice for the elliptic solver cycle—algorithmic steps 2 to 4—that is left at single precision. The consequence of this choice is that the latitude‐wise *L*_2_‐norms of the final residual error fields show a reduction of the convergence rate near the poles. The error is smaller (about an order of magnitude) when compared to the results from Figure 8d, but still clearly visible. The reduced solver convergence at the poles is however not causing an increase of the initial residual error fields near the poles. Thus one can conclude that the rest of the mixed‐precision shallow‐water model time‐step is not affected, possibly also due to the presence of the polar absorber.

The elliptic solver convergence of the resulting mixed‐precision shallow‐water model is shown in Figures 5e and 5f and 8e and 8f. The global, as well as latitude‐wise, representation of the solver convergence show that much of the behavior of the DP model variant is restored. Figure 8e shows that there is no accumulation of numerical errors at the poles anymore for the orographic wave test‐case (RHW4 not shown but qualitatively the same). The convergence rate at the poles is slightly reduced, see Figure 8f).

#### 4.2.1 Reformulation of the Initial Residual *r*_0_ Calculation

Based on Figure 9, a straightforward single precision calculation of *r*_0_ was shown insufficient. In the previous section, DP was instead used for this calculation. However, since the calculation of *r*_0_ is a computationally expensive operation, there is a strong incentive to explore further precision reduction via other means.

Rather than using higher precision, for some Krylov subspace methods a popular option to manage the accumulation of round‐off errors in the residual error field is a complete recalculation of the residual after a set number of solver iterations (Van Der Vorst & Ye, 2000). However, each recalculation adds another application of the linear operator L to the total cost of the solver. In our setup we only encounter up to 4 solver iterations until solver convergence, which renders a complete recalculation of the residual values to be computationally inefficient. Additionally, if the numerical errors in *r*_0_ become as large as the value itself near the poles, recalculation of *r*_0_ becomes meaningless.

Here, a different direction is explored. The numerical errors in *r*_0_ ultimately result from the truncation of the fluid thickness field Φ to single precision. It is presented how splitting parts of the residual calculation into the form of a base part plus a correction term can significantly reduce the numerical error in *r*_0_. Since the operator L is linear by design reformulating the calculation of *r*_0_ can be done with minimal effort.

As a preparation for splitting the operator calculation, first the fluid thickness variable Φ is split into two parts. Instead of a DP variable *Φ*, the fluid thickness field is now chosen to be described by two single precision fields: $\Phi \approx \hat{\Phi }+\tilde{\Phi }$, where $\tilde{\Phi }$ denotes a correction to some to‐be‐defined base fluid thickness field $\hat{\Phi }$.

At the beginning of the simulation, $\hat{\Phi }$ and $\tilde{\Phi }$ are set via the following procedure:

1. $\hat{\Phi }$ is set to the initial fluid thickness truncated to single precision

(16) $$\hat{\Phi }=trunc\left(\Phi^{0}\right).$$

2. $\tilde{\Phi }$ is then defined as the difference calculated in DP arithmetic between $\hat{\Phi }$ and Φ^0^ truncated to single precision

(17) $$\tilde{\Phi }=trunc\left(\Phi^{0}-\hat{\Phi }\right).$$

The average value of $\hat{\Phi }$ is then on the order or 10^4^–10^5^ m, while the average value of $\tilde{\Phi }$ is 10^−3^ m—a difference of 7–8 orders of magnitude which is consistent with the machine epsilon of single precision. The relative error of the sum $\hat{\Phi }+\tilde{\Phi }$ when calculated in DP arithmetic is on the order of 10^−15^ which is consistent with the DP machine epsilon.

During the simulation, the solver increments to fluid thickness are added to $\tilde{\Phi }$ each time‐step. Thus, the average values of $\tilde{\Phi }$ increase in time which increases the relative error in $\tilde{\Phi }$ and by definition also of the sum $\hat{\Phi }+\tilde{\Phi }$. After about 100 time‐steps, the average values of $\tilde{\Phi }$ are on the order of 10^1^ m, and the relative error of the sum $\hat{\Phi }+\tilde{\Phi }$ performed in DP arithmetic increases to about 10^−11^. If this continued further, eventually the relative errors of the sum $\hat{\Phi }+\tilde{\Phi }$ would reach the single precision machine epsilon.

As a result, in our approach we decide to update $\hat{\Phi }$ after every 100 time‐steps. This is a good compromise between precision and computational overhead. The procedure to update $\hat{\Phi }$ and $\tilde{\Phi }$ works as follows:

1. Temporarily store the sum

(18) $$\Phi^{DP}=\hat{\Phi }+\tilde{\Phi }$$

in a DP variable Φ^*DP*^

2. $\hat{\Phi }$ is set to the initial fluid thickness truncated to single precision

(19) $$\hat{\Phi }=trunc\left(\Phi^{DP}\right).$$

3. $\tilde{\Phi }$ is then defined as the difference calculated in DP arithmetic between $\hat{\Phi }$ and Φ^0^ truncated to single precision

(20) $$\tilde{\Phi }=trunc\left(\Phi^{DP}-\hat{\Phi }\right).$$

Since this update happens so rarely throughout the model simulation, the computational overhead is negligible.

So far, the split of a DP variable into two single precision variables has not resulted in any computational gains. Also, simply splitting the linear operator into a sum of two parts $L\left(\hat{\Phi }\right)+L\left(\tilde{\Phi }\right)$ does not offer any computational advantage as both parts would need to be recomputed every time‐step as the coefficients of the linear operator L change from one model time‐step to the next.

The trick to make this approach computationally efficient lies in a reformulation of the gradient calculation of *Φ*, that is, the partial derivatives $\frac{\partial \Phi }{\partial x^{J}}$, in the linear operator L of definition 12. The rewritten linear operator reads as follows:

(21) $$L\left(\Phi \right)≔\sum_{I=1}^{M}\frac{\partial }{\partial x^{I}}\left(\sum_{J=1}^{M}A^{IJ}\left(\frac{\partial \hat{\Phi }}{\partial x^{J}}+\frac{\partial \tilde{\Phi }}{\partial x^{J}}\right)+B^{I}\Phi^{SP}\right)-C\Phi^{SP}\;.$$

In this, $\Phi^{SP}=\hat{\Phi }+\tilde{\Phi }$ is the fluid thickness in single precision. This means, our approach requires a third single precision variable for fluid thickness. The $\partial \hat{\Phi }/\partial x^{J}$ only change every 100 time‐steps. Thus, they can either be precomputed and stored, which would result in two more single precision variables, or they could be recomputed every timestep if memory is crucial. Additionally, compared to the original definition 12, the operator (21) consists of two more floating point operations per grid‐point—the addition of the partial derivatives—but each of these is now performed in single precision arithmetic.

To sum up the required computations for the implemented versions of both operators. Each time‐step, the original operator requires 19 DP floating point operations per grid point and 10 DP variables. In comparison, the operator (21) consists of 22–24 single precision floating point operations and 12–15 single precision variables, depending on whether $\partial \hat{\Phi }/\partial x^{J}$ is stored of recomputed. Additionally, every 100th time‐step, there is an additional cost of four DP floating point operations.

**Figure 11:**
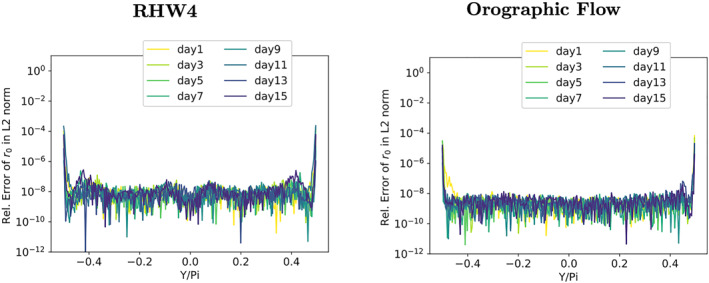
Latitude‐wise relative numerical error of the initial residual *r*_0_ calculated with the operator (21) with respect to the original operator (21) in double precision arithmetic in the *L*_2_‐norm for the RHW4 (left) and the orographic flow test‐case (right) in 2‐day time interval snapshots; darker colors indicate later simulation time.

Comparing Figure 9 and the latitude‐wise relative numerical errors when *r*_0_ is calculated using the single precision operator (21), see Figure 11, the relative numerical error is strongly reduced.

Using the new operator (21) in conjunction with the mixed‐precision model of Section 4.2 reveals that using the new operator throughout the entire computational domain is not desirable. This is because, although the relative error in each calculation of the initial residual error field *r*_0_ is greatly reduced near the poles, the remaining errors are exacerbated strongly due to the use of single precision in algorithmic steps 2–4. This leads to a vastly reduced convergence rate near the poles, with the latitude‐wise *L*_2_ − norm of the final residual errors being up to 10^−1^ for both test‐cases, looking very similarly to Figure 8d) that was found for the full single precision shallow‐water model variant. As a consequence, the latitude‐wise *L*_2_‐norm of the initial residual error field *r*_0_ near the poles can be as much as one order of magnitude larger than the values found for the mixed‐precision shallow‐water model of Section 4.2, see again Figure 8e).

To avoid this accumulation of numerical errors near the poles, from hereon the standard operator (12) is instead used for a three zonal band wide range closest to the North‐ and Southpole respectively, where all calculations are performed in DP. Model behavior is then completely recovered to that of the mixed‐precision shallow‐water model of Section 4.2.

### 4.3 Mixed‐Precision With Half Precision for the Elliptic Solver

In this section the aim is to take down precision to half precision for as many parts of the elliptic solver as possible while still satisfying a reasonably high solver accuracy.

The initial residual error *r*_0_ calculation in algorithmic step 1 is not considered for half precision arithmetic as the machine epsilon of half precision is 9.77 ⋅ 10^−4^ which means it is far larger than our targeted solver accuracy.

The focus is here primarily on the preconditioning and the application of the linear operator (12) in algorithmic steps 1 and 3. This is in part motivated by the fact that these steps are by far the most computationally intensive parts of the elliptic solver. It is found that these parts of the solver can indeed be taken down to half precision for the most part. Two exceptions from this rule however need to be made. The first exception concerns the last operation of the linear operator (12) where *C*Φ is subtracted from the previous terms. This calculation is done in single precision because *C*Φ and the remaining terms of the linear operator (12) are found to be of similar size and the subtraction of both terms introduces large numerical errors if done in half precision. The second exception is the area close to the poles. Since the operator (12) is ill‐conditioned near the poles, sufficient precision is found to be critical in these regions. Thus, for grid‐points within a three zonal‐band wide range closest to the North‐ and Southpole all calculations of the preconditioner and the linear operator are performed in single precision.

In comparison to the preconditioning and the application of the linear operator, algorithmic steps 2 and 4 are computationally cheap in terms of the normalized runtimes, consisting only of global sums and variable updates. The large range in the normalized cost of step 4 is due to the size of the sum changing with *ν*. Concerning the updates of the residual error field *r*_*ν*+1_ and the accumulation of solver increments to fluid thickness in the variable ΔΦ_*ν*+1_, both are kept in single precision. Both require precision higher than the machine epsilon of half precision to satisfy the solver accuracy. Concerning the update of $L\left(p_{\nu +1}\right)$, experiments where the sum is calculated in half precision arithmetic immediately reduces the attainable solver accuracy to 4 orders of magnitude for both test‐cases. Additionally all the summands are only available in single precision, which makes single precision the preferred option. However, the sum to obtain *p*_*ν*+1_ can be performed in half precision.

Concerning the global sums for *β* and *α*_*l*_ in algorithmic steps 2 and 4, single precision is the straightforward choice for these calculations. This is simply because all of the involved input variables are single precision and truncating them to half precision first would likely result in a computational overhead. Rounding errors in the global sums were on the order of the single precision epsilon and thus no issue for the studied model resolutions.

**Figure 12:**
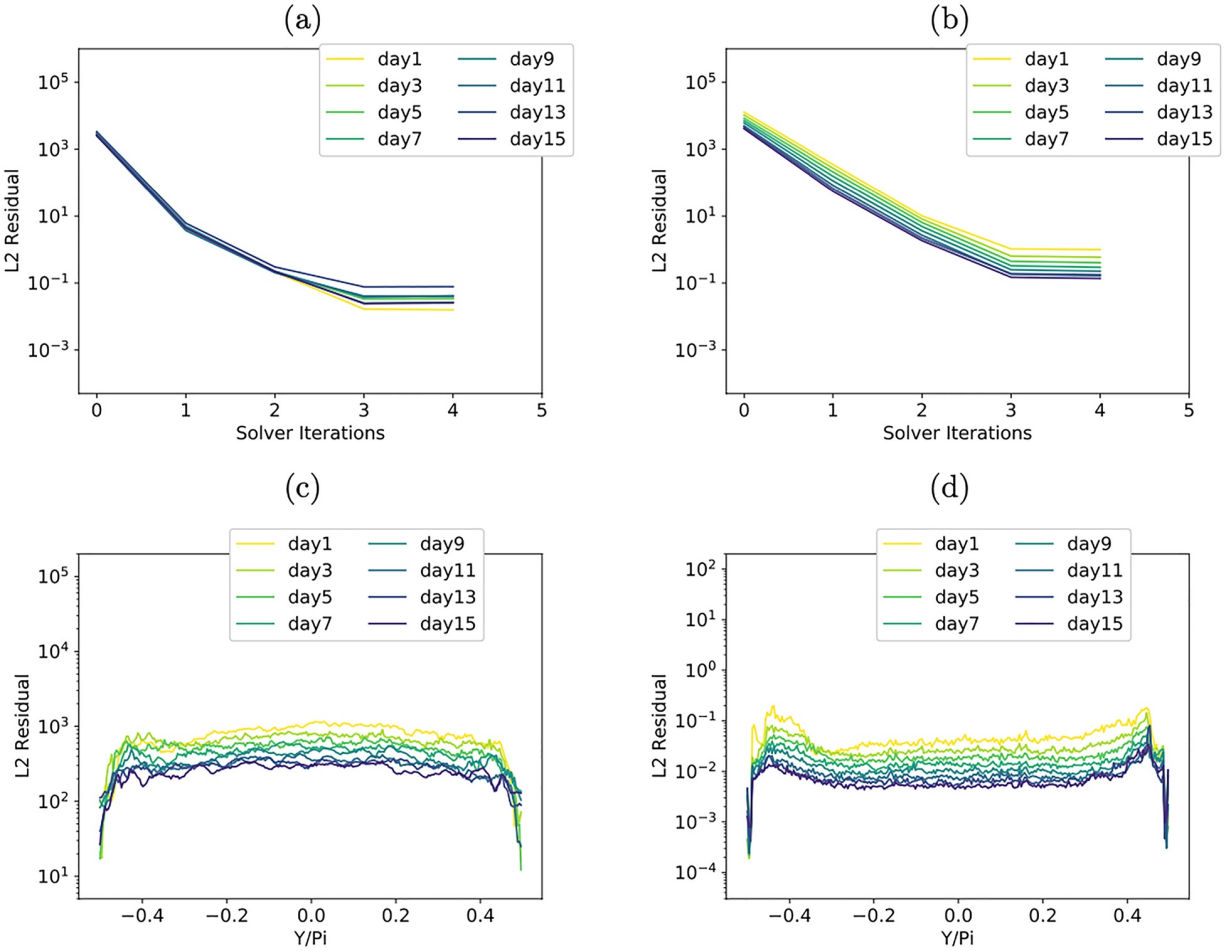
Convergence rates of the RHW4 (a) and the orographic flow test‐case (b) for the mixed‐precision elliptic solver variant with half precision, and the corresponding latitude‐wise initial (c) and final (d) residual error in the *L*_2_‐norm for the orographic flow test‐case.

The elliptic solver convergence of the resulting mixed‐precision shallow‐water model is illustrated in Figures 12a–12d. The global, as well as latitude‐wise, representation of the solver convergence show that much of the behavior of the DP model variant is preserved. But there is a clear degradation of solver convergence for the last solver iteration.

Figure 12c shows that there is no accumulation of numerical errors at the poles for the orographic wave test‐case. In Figure 12d, the impact of enforcing higher precision in the vicinity of the poles becomes visible. The final values for the latitude‐wise residual error *L*2‐norm near the poles are smaller in comparison to the rest of the computational domain, where the effect of low precision manifests in overall larger values when compared to previous results. The corresponding figures for the RHW4 are not shown but look qualitatively the same.

**Figure 13:**
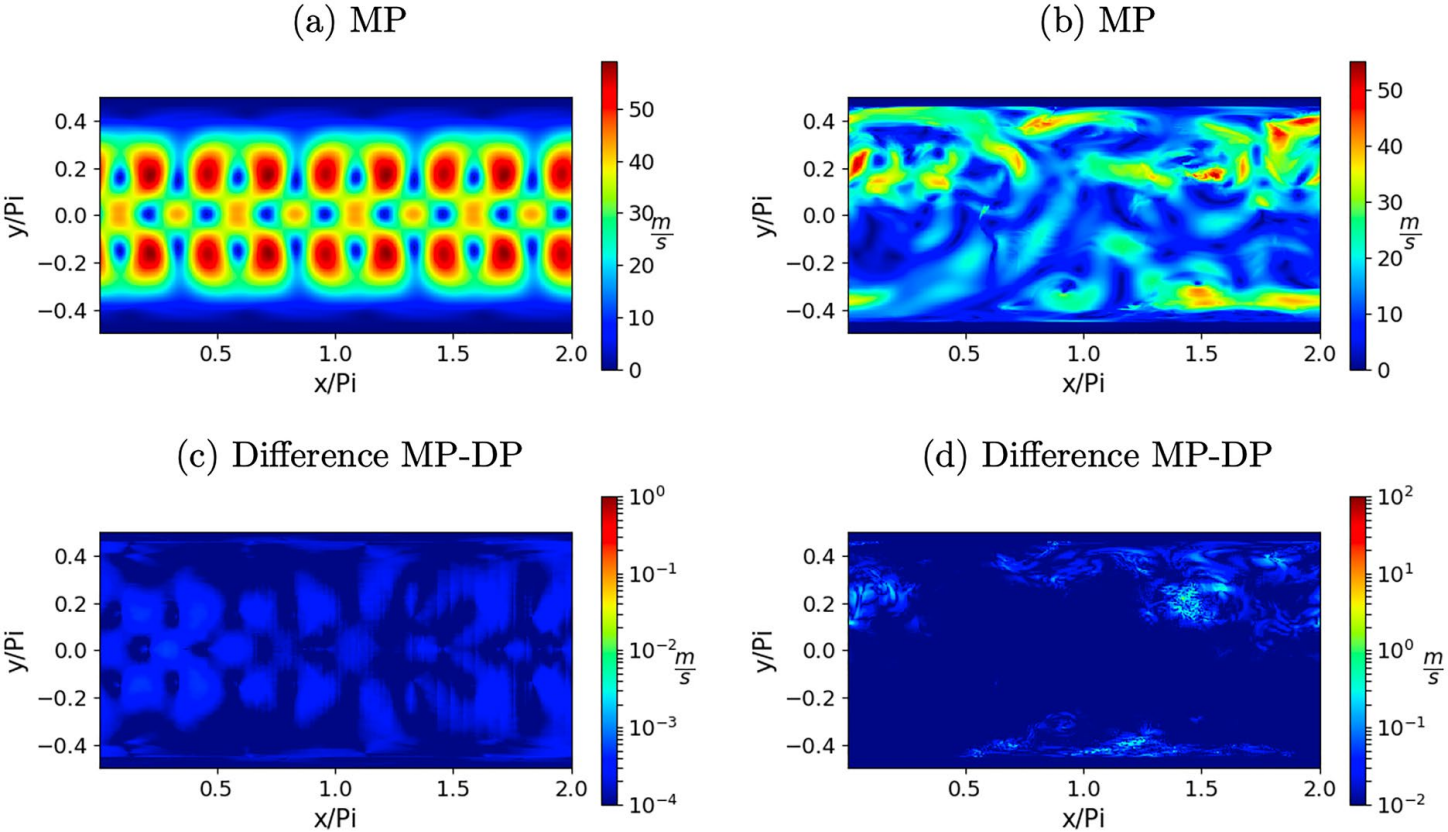
Square‐root of kinetic energy deviations in $\frac{m}{s}$ of the mixed‐precision (MP) model from the respective “genuine” model solution at *t* = 14.76 days for the RHW4 in (a), and the orographic flow test‐case in (b). Figures (c and d) show the respective difference in kinetic energy deviations between the double precision and mixed‐precision shallow‐water model solution in logarithmic scale.

Although the final residual error values are generally larger, the deviations from the genuine solution, which are shown in Figures 13a and 13b, are found to be very close to the DP shallow‐water model from Figures 6a and 6b. The differences in the deviations compared to the DP model, see Figures 13c and 13d, are at least one order of magnitude smaller than the difference found between single precision and DP from Figures 6e and 6f.

Coming back to Section 3.1, the two coarser model resolutions 256 × 128 and 128 × 64 were also run using the mixed‐precision shallow‐water model described in this section. As expected, it is found that the results are qualitatively the same (not shown here) and the analysis that has been done for the 512 × 256 applies as well to the coarser model resolutions. The vicinity near the poles for which higher precision levels are used is chosen to be two and one zonal band widths for the 256 × 128 and the 128 × 64 model resolution, respectively.

### 4.4 Expected Computational Performance of the Mixed‐Precision Solver

Ultimately, we are interested in increasing the solver's, and thus the entire model's, efficiency. For the simulations done in Sections 4.1 and 4.2, time measurements are performed to estimate the computational savings. The single precision shallow‐water model of Section 4.1 has a shorter time‐to‐solution than the DP shallow‐water model, the model runtime is reduced by 35%. The mixed‐precision shallow‐water model variant of Section 4.2 was 30% faster than the DP variant. For larger problem sizes, these differences between double and single precision performance are generally expected to become even larger. This is because larger problem sizes are typically limited by data transfer between memory, cache, and the processor, which is where low precision variables have a clear advantage as more relevant data can be stored closer to the processor. For instance, for state‐of‐the‐art W&C models single precision simulations are typically reporting a decrease of computing time by 40% in comparison to simulations in DP (Maynard & Walters, 2019; Váňa et al., 2017).

Concerning memory, the used memory is measured for the three different shallow‐water model variants. Memory usage is almost halfed when going from DP to single precision, which was expected since all variable are changed to single precision. For the mixed‐precision shallow‐water model variant of Section 4.2, memory was found to be 56%–60% of the memory used in DP.

For the mixed‐precision elliptic solver with half‐precision arithmetic (§4.3), the use of half precision is confined to the application of the preconditioner and the linear operator L. To study the impact of half precision on the performance of algorithmic step 3, the entire model code is ported to the Fujitsu A64FX, which supports half precision arithmetic from Fortran and is available on the Isambard 2 supercomputer. The model is compiled using the Fujitsu Fortran compiler 4.3.1 with the ‐kfz option. With the ‐kfz option, subnormal numbers are flushed to zero. Treating subnormal numbers differently on this chip would severely impact performance. Concerning variable rescaling, even though there is significant anisotropy in the linear operator L, after rescaling the coefficients are found to fit into the dynamical range of half precision of about 10 orders of magnitude. It was confirmed that the mixed precision variant on the A64FX provides the same results as the emulated reduced precision experiments in §4.3 in terms of the three criteria: convergence rate, solver accuracy and solution quality.

**Figure 14:**
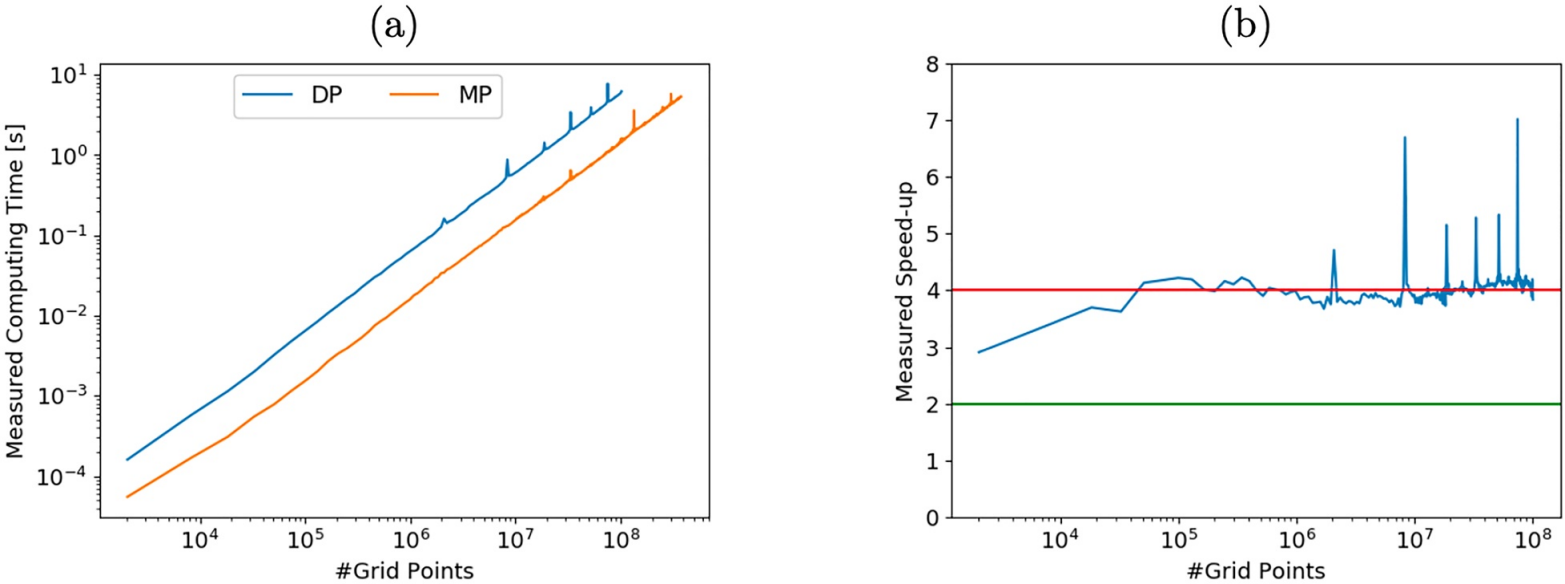
Measured runtimes (a) and Speed‐up (b) for algorithmic step 3 for the mixed‐precision variant (MP) compared to double precision (DP). The measured runtimes include necessary rescaling and changes in precision levels.

The measured runtimes for the mixed precision variant and the DP variant of algorithmic step 3 are shown in Figure 14a). These numbers include the rescaling of variables and the type‐casting between different precision levels. Time measurements are performed for a wide range of problem sizes until memory limits are reached. The mixed precision variant is consistently faster. Also, it uses only 27% of the memory i.e., required for DP. Speed‐ups, see Figure 14b), for larger grid sizes lie between 3.8 and 4.2 consistently. For more information on half precision performance on the A64FX for physical models, see (Klöwer et al., 2022).

To keep the porting to Isambard 2 simple, estimates for the total reduction in solver runtime will be based on the savings for algorithmic step 3. Given the measured speed‐ups for algorithmic step 3, it is justified to assume a speed‐up of factor of 4 for half precision and a respective factor 2 for a reduction to single precision level. The same holds for memory. These assumptions are in accordance with the peak performance of modern processors and are also justified by the increase of cache, memory and bandwidth efficiency. To estimate the total reduction of runtime, we multiply the number of floating point operations that has been performed with the three different precision levels (double, single and half) with their respective estimated cost (1.0, 0.5, 0.25) for the different algorithmic steps. Including the exceptions for precision levels that we made in §4.3, the overall runtime can be expected to reduce by a factor of 3.3, when comparing the mixed‐precision elliptic solver with half‐precision to the DP variant.

## 5 Conclusion

Mixed‐precision in a representative SI shallow‐water model in general and its preconditioned elliptic solver in particular is found to be possible and overall advantageous when compared to the default DP option. The described mixed‐precision shallow‐water model using double and single precision clearly outperforms the DP model in terms of time‐to‐solution while maintaining the same solution quality and similar solver convergence behavior. Concerning the elliptic solver, most of the computationally expensive components of the solver can be performed almost entirely in half precision arithmetic.

This being said, it is found that the special structure of our problem does not permit a naive reduction of precision. We should expect the exacerbation of artificial modes in the prognostic variable fields of the model when going to too low precision levels in the elliptic solver. In our configuration, precision reduction close to the poles—the two grid‐singularities that were present—caused spurious behavior even for the single precision shallow‐water model. Overall, as the solver precision is further and further reduced to its limits throughout this paper from DP to precision levels as low as half precision, a very clear progression is seen in terms of an increase in numerical errors, reduced convergence rates, and the increasing need for assigning higher precision levels for parts of the computational domain.

On the other hand, it is shown that given some knowledge about the specific structure of the application, issues with reduced precision can in fact be mitigated by using higher precision arithmetic in parts of the computational domain. The path toward a computationally efficient mixed‐precision elliptic solver can be a challenging one though, as it requires a holistic way of thinking about the interplay between numerical errors from a local precision reduction, to the resulting global model errors, and the solver's overall convergence rate. Understanding this interplay may sometimes require in‐depth analysis and in some cases even the reformulation of parts of the existing solver algorithm. The approach we found might also be beneficial in other applications.

All in all, results are similar to (Amritkar & Tafti, 2016; Anzt et al., 2019; Baboulin et al., 2009; Carson & Higham, 2018; Furuichi et al., 2011; Göddeke et al., 2007; Haidar et al., 2018; Idomura et al., 2018) in that the preconditioning step is very robust against reducing precision and seems to be the part of the elliptic solver where performance gains can be expected while many of the other parts can be quite sensitive to reducing precision. It is an important finding that this general structure can also be found in our specific problem from geophysical fluid dynamics, using a conjugated residual solver with a preconditioner based on tridiagonal inversion, a key component in 3D atmospheric SI grid‐point models.

A recurrent theme in previous publications on reduced precision in W&C models is the motivation that there are irreducible uncertainties (such as initial condition uncertainties, stochastic representations of unresolved subgrid‐scale processes) present in these models which gives a justification for accepting a certain amount of additional model errors, in the form of reducing precision, in order to increase the overall model's computational efficiency (Chantry et al., 2019; Düben et al., 2014; Hatfield et al., 2018; Palmer, 2012; Saffin et al., 2020). It should be noted that in the elliptic solver this idea of accepting a certain amount of additional model error to increase computational efficiency is at the very core of the approach and is inherently represented and used in the process of setting the solver accuracy.

In summary, given our results, we do not see any major obstacle that would prevent us from using reduced precision arithmetic for an elliptic solver applied to a full 3‐dimensional atmospheric model. There is much experience within the atmospheric modeling community concerning the behavior of the height/pressure solve via iterative methods on various computational grids which can be used to circumvent possible pitfalls when reducing precision and instead guide the path toward efficient mixed‐precision elliptic solvers for W&C models.

## Data Availability

No primary data were used for this study. The source code that was used to produce the results of the paper is available at https://github.com/JanAckmann/RP_Semi_Impl_SW.

## Appendix A Coefficients of the Linear Operator

The derived coefficients of the linear operator (12) are presented. In this, the coefficients are indexed by gridpoints that are denoted by *I* = 1…*N* in longitudinal direction and *J* = 1…*M* in meridional direction.

The *A*‐components:

$$A^{11}\left(I,J\right)=-g\left(\Phi^{n}\left(I,J\right)-H_{0}\left(I,J\right)\right)\;MGI_{hx}\left(I,J\right)\;A\left(I,J\right)\;C\left(I,J\right),$$

$$A^{12}\left(I,J\right)=-g\left(\Phi^{n}\left(I,J\right)-H_{0}\left(I,J\right)\right)\;MGI_{hy}\left(I,J\right)\;B\left(I,J\right)\;C\left(I,J\right),$$

$$A^{21}\left(I,J\right)=-g\left(\Phi^{n}\left(I,J\right)-H_{0}\left(I,J\right)\right)\;MGI_{hx}\left(I,J\right)\;B\left(I,J\right)\;D\left(I,J\right),$$

$$A^{22}\left(I,J\right)=-g\left(\Phi^{n}\left(I,J\right)-H_{0}\left(I,J\right)\right)\;MGI_{hy}\left(I,J\right)\;A\left(I,J\right)\;D\left(I,J\right),$$

the *B*‐components:

$$B^{1}\left(I,J\right)=-E1\left(I,J\right)\;A\left(I,J\right)\;C\left(I,J\right)-E2\left(I,J\right)\;B\left(I,J\right)\;C\left(I,J\right),$$

$$B^{2}\left(I,J\right)=-E2\left(I,J\right)\;A\left(I,J\right)\;D\left(I,J\right)+E1\left(I,J\right)\;B\left(I,J\right)\;D\left(I,J\right),$$

and the *C*‐component:

C(I,J)=1,

where:

$$MGI_{hx}\left(I,J\right)=-\frac{GH1}{h_{x}\left(I,J\right)},\;MGI_{hy}\left(I,J\right)=-\frac{GH2}{h_{y}\left(I,J\right)}.$$

$$A\left(I,J\right)=\frac{1}{1+GMM{\left(I,J\right)}^{2}}$$

B(I,J)=A(I,J)GMM(I,J)

$$C\left(I,J\right)=0.5\;GH1\;h_{y}\left(I,J\right)$$

$$D\left(I,J\right)=0.5\;GH2\;h_{x}\left(I,J\right)$$

$$E1\left(I,J\right)=-\frac{GH1\;g\left(\Phi^{n}\left(I+1,J\right)-\Phi^{n}\left(I-1,J\right)\right)}{h_{x}\left(I,J\right)}$$

$$E2\left(I,J\right)=-\frac{GH2\;g\left(\Phi^{n}\left(I,J+1\right)-\Phi^{n}\left(I,J-1\right)\right)}{h_{y}\left(I,J\right)},$$

with:

GMM(I,J)=0.5Δtf(J)

$$h_{x}\left(I,J\right)=a\;\cos\left(\phi \left(J\right)\right),\;h_{y}\left(I,J\right)=a$$

$$GH1=0.5\frac{\Delta t}{\Delta x},\;GH2=0.5\frac{\Delta t}{\Delta y}.$$
